# Robust network topologies for generating oscillations with temperature-independent periods

**DOI:** 10.1371/journal.pone.0171263

**Published:** 2017-02-02

**Authors:** Lili Wu, Qi Ouyang, Hongli Wang

**Affiliations:** 1 The State Key Laboratory for Artificial Microstructures and Mesoscopic Physics, School of Physics, Peking University, Beijing, China; 2 Center for Quantitative Biology, Peking University, Beijing, China; 3 Peking-Tsinghua Center for Life Sciences, Peking University, Beijing, China; Georgia State University, UNITED STATES

## Abstract

Nearly all living systems feature a temperature-independent oscillation period in circadian clocks. This ubiquitous property occurs at the system level and is rooted in the network architecture of the clock machinery. To investigate the mechanism of this prominent property of the circadian clock and provide general guidance for generating robust genetic oscillators with temperature-compensated oscillations, we theoretically explored the design principle and core network topologies preferred by oscillations with a temperature-independent period. By enumerating all topologies of genetic regulatory circuits with three genes, we obtained four network motifs, namely, a delayed negative feedback oscillator, repressilator, activator-inhibitor oscillator and substrate-depletion oscillator; hybrids of these motifs constitute the vast majority of target network topologies. These motifs are biased in their capacities for achieving oscillations and the temperature sensitivity of the period. The delayed negative feedback oscillator and repressilator are more robust for oscillations, whereas the activator-inhibitor and substrate-depletion oscillators are superior for maintaining a temperature-independent oscillation period. These results suggest that thermally robust oscillation can be more plausibly achieved by hybridizing these two categories of network motifs. Antagonistic balance and temperature insulation mechanisms for achieving temperature compensation are typically found in these topologies with temperature robustness. In the temperature insulation approach, the oscillation period relies on very few parameters, and these parameters are influenced only slightly by temperature. This approach prevents the temperature from affecting the oscillation period and generates circadian rhythms that are robust against environmental perturbations.

## Introduction

Robustness against environmental perturbations, particularly ambient temperature variations, is a key property of living systems. Thermal robustness has been reported recently in the signaling process of bacterial chemotaxis in *E*. *coli* [1] and in Notch signaling in the development of *Drosophila* [2]. A prominent and intensively investigated example of thermal robustness is temperature compensation in circadian clocks; circadian clocks are ubiquitous in life forms from bacteria to humans [3–5]. Despite temperature changes, circadian clocks maintain endogenous and robust rhythmic activities with a period of approximately 24 hours in harmony with the environmental daily rhythm. A temperature-independent period and entrainment by zeitgebers are two fundamental qualities of circadian clocks. Over the past two decades, the molecular basis of circadian clocks, which is generally a network of transcription-translation feedback loops [3, 6, 7], has been delineated using model organisms [8–10]. Several explanations for the phenomena of temperature compensation have been proposed. A popular and mathematically natural mechanism is antagonistic balance [11–21], in which the temperature-independent period is achieved by a delicate balance that requires fine-tuning of parameters. To account for robustness to mutations in circadian clock genes and, consequently, changes in kinetic rate constants and activation energies, a switch-like mechanism has been proposed [22]. Another scheme without the need for fine-tuning parameter values was proposed for systems with several reactions catalyzed by a common enzyme, in which the temperature compensation is based on an enzyme-limited mechanism [23, 24]. A recent notable explanation attributed compensation to an adaptation that buffers temperature changes [25, 26] via a temperature-insensitive core oscillator coupled to a specific adaptive temperature signaling pathway.

These explanations for the mechanism of circadian clocks have explained temperature compensation at the system level. As supported by experimental evidence, the temperature-independent oscillation period is most likely a system-level property [24, 27]. In addition to the influence of nonlinearity in reaction kinetics, this remarkable property could also be rooted in the network architecture of elementary steps and feedback loops that consist of the circadian clock. As most biochemical reactions must overcome an energy barrier *E*_*i*_ with the aid of enzymes, the reaction rate could be plausibly written in Arrhenius form, *k*_*i*_~exp(−*E*_*i*_/*RT*). Temperature compensation is mathematically described by the following antagonistic balance,

$$\frac{dlnP\left(T\right)}{dT}=\sum_{i}\frac{\partial lnP}{\partial lnk_{i}}\frac{dlnk_{i}}{dT}=\frac{1}{RT^{2}}\sum_{i}C_{i}E_{i}=0$$ (1)

where *C*_*i*_, defined as $\frac{\partial lnP}{\partial lnk_{i}}$, is the control coefficient obeying the summation theorem Σ_*i*_*C*_*i*_ = −1 [17], and *E*_*i*_ is the activation energy. Generally, the oscillation period *P* depends in an unknown way on all constants in the model. The activation energies are properties of the individual reaction steps. However, the control coefficients could be involved in the underlying reaction network as a whole. That is, temperature-compensated oscillators could depend strongly on the topologies of the network. The function of temperature compensation would impose constraints on the circuit topologies of the circadian oscillators, and there might be only a limited number of network topologies that are capable of robust temperature-compensated oscillations (TCOs). This raises the question of what core structures and design principles of biochemical oscillators featuring a temperature-independent period are commonly shared by circadian clocks. Philosophically, structure determines function, and the topology of networks is key to understanding their central properties [28–31]. The core topologies capturing the backbone of practically complex networks have been investigated for simple functions such as oscillations [32], adaptation [33] switch-like responses [34], dose-response alignment [35] and patterning in response to morphogen gradients [36].

In this paper, we intend to investigate the mechanism and design principles of oscillations with temperature-independent periods and to provide general guidance for designing genetic oscillators with this property. We consider theoretically simple genetic regulatory networks and perform a complete search for networks capable of oscillations with a temperature-independent period. We enumerate two- and three-node networks by imposing the constraints of oscillations and a temperature-independent oscillation period and focus on the core network topologies and design principles of TCOs. The function of temperature compensation shows a preference in the wiring diagram of the underlying networks. We find four network motifs, namely, a delayed negative feedback oscillator, repressilator, activator-inhibitor oscillator and substrate-depletion oscillator, whose hybrids constitute the vast majority of our targeted network topologies. Analyses show that most of the networks that can perform robust oscillations with temperature-compensated oscillation period are typically combinations of two types of core motifs that are complementary to each other, i.e., one type is more robust for achieving oscillation but is weaker for compensation, and the other type is more robust for having a temperature-independent oscillation period but is fragile for oscillations. An insulation mechanism for the temperature-compensated period is adopted generally: the oscillation period is controlled by very few parameters that are insensitive to temperature changes. This mechanism avoids changes in the oscillation period due to temperature variations and generates circadian rhythms that are robust against environmental perturbations.

## Results

### Searching networks capable of achieving a temperature-compensated oscillation period

A temperature-compensated oscillation period is defined by an oscillation period that remains constant when the temperature changes significantly. To gain insight into the core structure and design principle of TCO networks, we exhaustively enumerated all topologies with less than three nodes to identify genetic interaction circuits that could generate TCOs. Although practical circadian clocks featuring temperature compensation mostly have more than three nodes, they can be coarsely grained to simple networks with fewer nodes. We restricted this analysis to consider simple networks with three nodes due to the limitation of computational power and time restrictions for the exhaustive exploration of networks with more nodes.

We considered a total of 2423 topologically non-equivalent network topologies with two and three nodes (see Methods). Each node in the network represents a gene and its protein production. A directional link, → or ⊸, from one node to another denotes that the protein production of one gene regulates the expression of the other gene as a positive or negative transcription factor. Dynamical behaviors of the interacting genes were determined by rate equations in the form of a set of coupled ordinary differential equations [26, 37] that describe the time evolution of protein concentrations. The rate equation for a network node consists of three parts: the basal expression rate; the rate contributed by transcriptional factors; and the degradation rate due to proteases or increased cell size. Temperature effects were introduced theoretically into the dynamics by means of the Arrhenius law. We assume that all rate constants are temperature dependent [38, 39], and the corresponding activation energies are sampled uniformly in the range from 1KJ/mol to 100KJ/mol; the remaining parameters are temperature independent. The Arrhenius equation used here may provide a simplified estimate of the complicated temperature dependence of actual cellular (transcription and translation) processes.

We randomly assigned 10,000 sets of parameter combinations for each topology using the Latin hypercube sampling method [40]. For several simple topologies, random sampling has been expanded to 100,000. A certain topology and a set of parameters constitute a transcriptional regulation circuit. For each circuit, we first checked whether it is oscillatory. Subsequently, we assessed the TCO property of an oscillating circuit by calculating the relative standard deviation (RSD) of oscillation periods obtained at different temperatures in the range from 283K to 303K. Circuits with RSD below 10% were considered capable of TCO. To evaluate the overall performance of each topology, we chose to characterize its oscillation and TCO ability based on Q- and q-values, respectively. The Q-value was defined for a topology as the number of parameter sets from the total 10,000 sets that can maintain oscillations. Similarly, the q-value was defined as the number of parameter sets that can achieve TCO. The Q- and q-values are estimates of the volumes in the parameter space that allow oscillation and TCOs, respectively. These values were adopted individually as measures of robustness to achieve oscillations and the temperature compensation.

### Structural characteristics of TCO networks

#### Different topologies vary greatly in robustness to achieve TCO

Among all possible distinct topologies, more than half had at least one sampled set of parameters to maintain oscillations when temperature was scanned. From 1504 oscillatory topologies, we identified 787 distinct topologies with at least one set of parameter combinations to oscillate with temperature compensation. The q-values of these TCO topologies, which measure the ability to achieve TCO, vary greatly, and very few have relatively large q-values. The number of TCO topologies falls exponentially with increasing q-value (See S1 Fig). This pattern implies that the function of robust TCO might strongly depend on the network topology.

**Fig 1:**
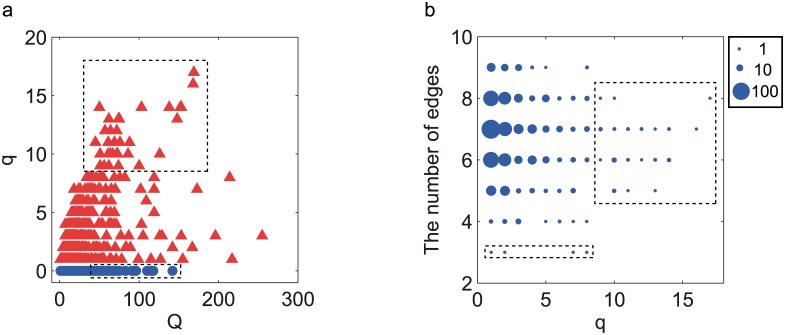
The overall performance of TCO networks. (a) Distribution of oscillatory topologies in Q-q-value space. Red triangles represent networks with at least one circuit with TCO, i.e., q-value > 0; blue circles are for oscillatory networks that do not have TCO parameters, i.e., q-value = 0. Robust topologies with top q-values (q-value > 8) are highlighted in the upper frame, which surrounds 35 networks that are best for TCO. There are another 35 “worst” topologies that are not thermally robust (q-value = 0) but have high Q-values (Q-value > 40), as marked by the lower frame. (b) The TCO topologies in (a) are re-plotted in the space of q-value and number of links (k). The circle size is proportional to the number of topologies with a specific (q, k) combination. The best TCO topologies are highlighted again in the upper frame. Topologies with the least number of links (k = 3) are emphasized in the low square.

The overall searching results are summarized in Fig 1. Fig 1a demonstrates the distribution of oscillatory topologies in the Q-q space. The majority of the topologies aggregate on the left-bottom with small Q- and q-values. Only a small number have a relatively larger Q- or q-value, indicating that there are few topologies robust for generating oscillations (large Q-value) or robust for TCOs (large q-value). For further examination, we selected the best and worst topologies for TCO in the Q-q space. As highlighted in the upper frame in Fig 1a, 35 networks with q-values greater than 8 are recognized as best for TCO. These networks have a relatively larger fractional parameter space for TCO. As denoted in the low frame, there are 35 networks with Q-values greater than 40 and a zero q-value, which are considered “worst” for TCO. These networks oscillate easily but encounter difficulty in finding parameter combinations to achieve temperature compensation.

Fig 1b shows the 787 TCO topologies distributed in the space of q-value and the number (k) of links. The q-k combinations for the best TCO topologies depicted in Fig 1a are denoted by the upper frame. The topologies that are robust for TCO (with q-value > 8) tend to have 5 to 8 edges. In particular, the 4 simplest topologies (highlighted in the low frame) have the least number of links. These simplest topologies with three links are of special interest because they are the minimal topologies that can achieve TCO. As will be shown in the following, these topologies are actually the core topologies or TCO motifs that combine to form complex and robust TCO networks.

#### Structure decomposition of TCO networks

**Fig 2:**
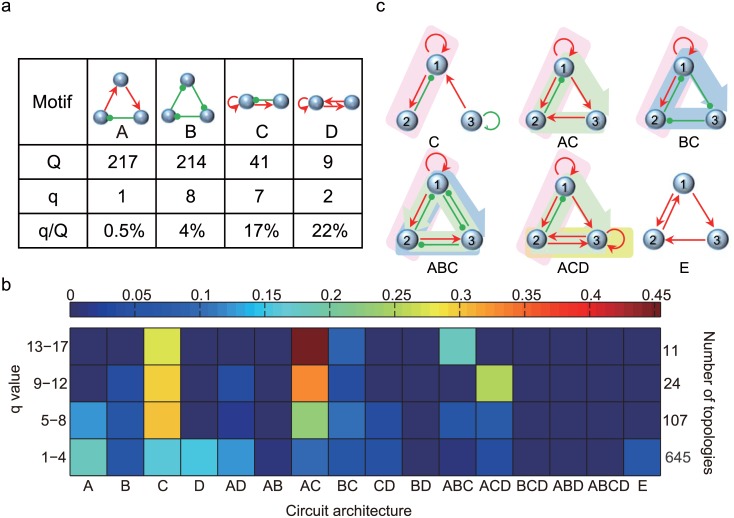
Structure decomposition of TCO networks. (a) The simplest network motifs with TCO, namely, delayed negative feedback oscillator (motif A), repressilator (motif B), activator-inhibitor (motif C) and substrate-depletion (motif D). Motifs A and B have relatively larger Q but smaller q/Q ratio compared with motifs C and D. (b) The distribution of TCO topologies in the q-value-and-motif-combination space coded by color. TCO topologies that do not contain any of motifs A, B, C, or D are classified as E-class (shown at the right end of the horizontal axis). The sum of topologies in each q-value range is shown on the right. The percentage of each type of circuit architecture in the corresponding q-value range is coded in color. Due to structural conflict in regulations, motif B and motif D cannot coexist in a three-node network so that combinations BD, ABD, BCD, ABCD (and similarly motif A and motif B combinations) are not permitted. (c) Several examples of TCO topologies composed of simple motifs or their combinations: C, BC, AC, ABC, ACD, together with one that falls in the E-class. The green, blue, pink and yellow areas denote motifs A, B, C and D, respectively.

The four simplest TCO topologies with the least number of edges are depicted in Fig 2a. Notably, these topologies are classical network motifs for oscillations: motifs A and B are the simplest negative feedback loops with three components and are called the delayed negative feedback oscillator [41] and repressilator [42], respectively. Both are classic mechanisms for periodic protein expression. Motifs C and D are the simplest two-component oscillators based on autocatalysis and are called the activator-inhibitor oscillator and substrate-depletion oscillator [43], respectively. While each of the simplest motifs yields oscillations, they differ drastically in their capacity for achieving temperature compensation. Fig 2a shows that motifs A and B have obviously large Q-values but relatively small q-values. By contrast, the Q-values of motifs C and D are much smaller, but the q-values of these motifs are comparable with those of motifs A and B. To differentiate the basic motifs A, B, C, and D, we adopt the ratio q/Q as a measure of their ability to achieve temperature compensation in the premise of oscillations. The relative value q/Q is used because TCOs are first oscillatory. Fig 2a depicts that the ratios for motifs A and B (0.5% and 4%, respectively) are significantly smaller than for motifs C and D (17% and 22%, respectively). The data were recalculated by sampling tenfold additional parameter sets, and ratios of 1.76%, 6%, 16% and 18% were obtained individually for motifs A, B, C, and D. Based on these observations, the basic motifs can be roughly classified into two categories: one category that includes motifs A and B, which can readily achieve oscillations but are weak at achieving compensation; the other category is composed of motifs C and D, which are more thermally robust for temperature compensation in the premise of oscillation. Motif combinations of these two categories can reasonably yield topologies that are robust in TCOs (lager q-values).

Motifs A, B, C, and D play an essential role in the construction of TCO topologies. Fig 2b demonstrates the distribution of TCO topologies in the q-value-and-motif-combination space, which shows that the largest majority of TCO topologies adopt at least one of the four motifs or their combinations as their core structures. In Fig 2b, the q-value axis is coarse-grained into four intervals (i.e., the ranges 1–4, 5–8, 9–12, and over 12), and the total number of networks within each q-value range is shown on the right side. There are 16 types of motifs or motif combinations listed on the horizontal axis. A topology that contains motif A without motif B, C or D is classified as A-class, and a topology that contains motif A and B without motifs C and D is categorized as AB-class, etc. A small fraction (approximately 5%) of the 787 TCO topologies does not contain any of the four simplest motifs and are classified as E-class. The color represents the percentage of core structure type in the total 16 types within each q-value range. The percentage is obtained by dividing the number of network topologies with one of the 16 types of core structures by the total number of topologies whose q-value falls in the specific range. For example, 3 topologies only consist of motif C with q > 12, and 11 topologies have q-values larger than 12; the “color” is therefore just the fraction 3/11 (about 27%).

From the perspective of structure composition, robust TCO networks prefer combinations of motifs from categories A, B and C, D, particularly the combination of motifs A and C. Motifs A, C and D are abundant in TCO topologies within the lowest q-value range. Among the TCO networks that we find, motif C plays a special role. Numerical examinations of TCO data for motif C reveal that the oscillation period is constrained to be sensitive to a few constants that are not affected significantly by temperature changes because they have low activation energies (A detailed analysis will be addressed later). This mechanism for TCOs could be preserved in networks containing motif C, particularly the combination with motif A, which is most robust for oscillations. As depicted in Fig 2b, motif C and its hybrids with motifs A, B, such as AC, ABC, ACD, are prominent in the relatively high q-value range (with qit). Other combinations, such as AD, BC, CD, also appear in TCO networks but have only small proportions. At first sight, the BC combination would be superior to the AC combination because motif B has even better q-value than motif A as depicted in Fig 2a. This is in contrast with the results in Fig 2b where the AC combination is more abundant than the BC combination. Checking into the network structures reveals that the AC combination is advantageous because the means for combining motif B and motif C is inherently less than that for AC combinations due to the special successive repression structure in motif B. That is, motif A and motif C can be much more flexibly combined than the BC combination. This makes the AC combination more abundant and advantageous. In the simplest networks of AC and BC combinations (S2 Fig), there are three distinct AC combinations but with one BC combination. The advantage of AC combination over BC combination can be more clearly seen in the networks with high q-value (q > 12) (S3 Fig). Fig 2c demonstrates several robust TCO topologies backboned with motif hybrids of A, B and C, D, together with a TCO topology of E-class, which could be essentially a variant of motif D.

#### Structure comparison between the best and worst TCO networks

**Fig 3:**
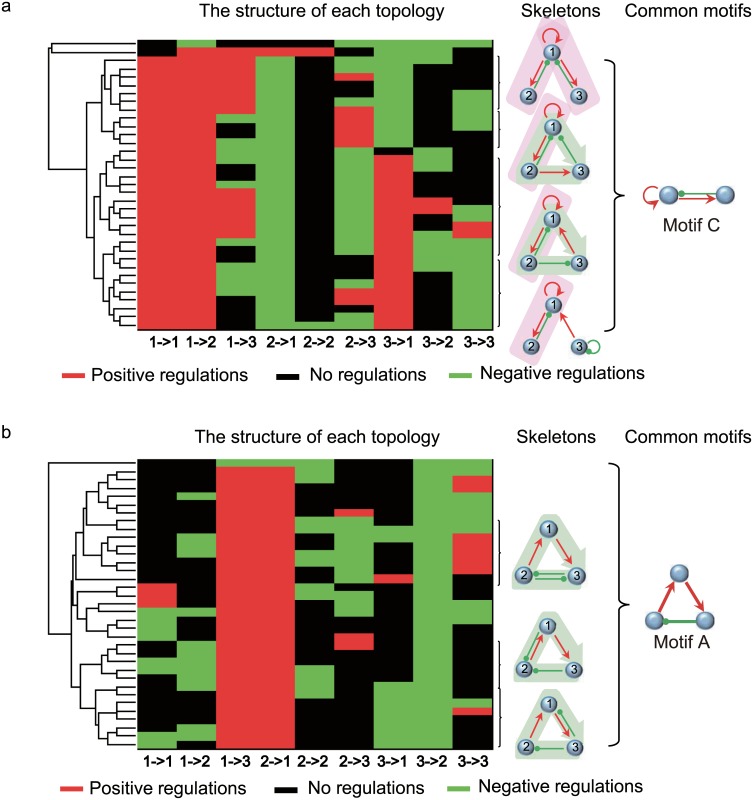
Clustering of best and worst oscillatory networks for temperature compensation. (a) Clustering of 35 TCO networks with top q-values. (b) Clustering of 35 worst TCO networks with top Q-values but a zero q-value. Positive, negative and null regulations between the nodes are denoted by red, green and black, respectively. Each row demonstrates the interaction combination between Nodes 1, 2, 3 in a network. The skeletons depict typical network topologies and motif constituents as revealed in the clustering of both the best and worst TCO topologies, respectively. The right motifs C and A show individually the most common core structures in the two categories of networks.

Because we checked the simulation data of these topologies with high Q- but zero q-values, the period susceptibility ∂*lnp*/∂*lnk*_*i*_·∂*lnk*_*i*_/∂*T* is dominantly negative, i.e., an increase in temperature accelerates the oscillation and decreases the oscillation period (see S4 Fig). By contrast, for the best TCO networks with high q-values, the positive and negative susceptibilities of rate constants are equally balanced. We consider the best 35 TCO topologies with top q-values and the worst 35 oscillatory networks with a zero q-value but highest Q-values (see Fig 1) and focus on the structural features that differ between these two categories of networks. The worst TCO topologies encounter difficulty in achieving antagonistic balance as expressed in Eq 1. Fig 3 depicts the analyses of structure clustering. In the clustering of the top TCO topologies (Fig 3a), the regulations commonly shared by this category of networks include the positive self-activation of node 1 (1act and the negative feedback loop between node 1 and node 2 (1→2, 2⊸1), which constitutes motif C. In addition, motif A, which is a negative feedback loop among the three nodes, is also most abundant in the best TCO networks. By contrast, clustering of the “worst” TCO networks (Fig 3b) shows that motif A is the sole backbone globally shared in the worst TCO networks. The results indicate that the chief difference between the best and worst networks is whether motif C appears in the network topology or not.

The clustering analysis agrees with the results in Fig 2a: motif A is the core motif for achieving robustness in oscillations, whereas motif C is the key factor for enhancing the thermal robustness of oscillations. These two core motifs can combine to produce stable oscillations with a period robust to temperature changes. To achieve simply the goal of oscillation, it is better to choose motif A as the core structure because its Q-value is large; however, for robust TCOs, motif C or its hybrid with motif A is recommended as the backbone of the oscillators.

### Mechanism of temperature compensation

**Fig 4:**
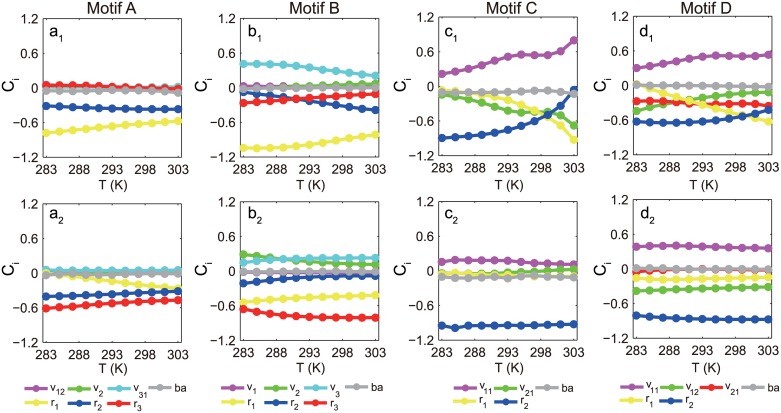
Control coefficients for simple TCO motifs. The temperature dependence of the control coefficient *C*_*i*_ (defined as ∂*lnP*/∂*lnk*_*i*_), which measures the parameter sensitivity of the oscillation period, is depicted for motifs A (*a*_1_, *a*_2_), B (*b*_1_, *b*_2_), C (*c*_1_, *c*_2_) and D (*d*_1_, *d*_2_), respectively, with each topology having two sets of parameters for TCOs. The parameter values used in our calculation of these TCOs are provided in the S1 Text.

The realization of temperature-compensated oscillation relies on the satisfaction of Eq 1 over a wide temperature range. From the general condition, TCOs depend on two key factors, i.e., the control coefficient *C*_*i*_ (which can be either positive or negative) and the activation energy *E*_*i*_ (which is always positive). *C*_*i*_ depends on temperature in a complex manner, which complicates mathematical analysis of TCOs. To investigate the mechanisms of TCOs, we calculated the control coefficients *C*_*i*_s and activation energies *E*_*i*_s for all TCO circuits. The *C*_i_~*T* dependences would be very different for different realizations of TCOs, even for the same TCO topology. Fig 4 illustrates the control coefficients as a function of temperature when TCOs are realized with the basic motifs A, B, C, and D. The pairwise comparison in the upper and lower rows for these motifs illustrates that the *C*_i_~*T* dependences are diversified. More examples of TCO and non-TCO examples for motifs A, B, C, and D and hybrid topologies are illustrated in S5 and S6 Figs in the Supporting Information.

Theoretically, the balance of Eq 1 can be achieved in different ways. The simplest is to have zero or near-zero activation energies for all reactions. This situation is trivial because the TCO has no structural preference. A general case would be that the oscillation period depends extensively on the reaction constants and that the reaction rates are sensitive to temperature changes (i.e., with large *C*_*i*_*s* and *E*_*i*_*s*). With increments in the temperature and thus in the reaction constants, the oscillation is either typically accelerated by the rates with negative *C*_*i*_*s* or hindered atypically by those with positive *C*_*i*_*s*. A balance is delicately maintained between the two opposing effects to ensure a temperature-independent oscillation period. In this case, the antagonistic balance imposes constraints on the parameters and is called distributed temperature compensation [18]. This antagonistic balance was observed in the TCO topologies we screened. As illustrated in panels b_1_, b_2_, c_1_, d_1_, and d_2_ of Fig 4, TCOs were realized antagonistically by proper combinations of activation energies.

The second mechanism is the temperature insulation scheme. In this situation, reactions with control coefficients of large amplitude can strongly impact the oscillation period. These reactions are insensitive to the temperature due to their small activation energies, which prevents temperature changes from affecting the oscillation period. In this case, other parameters could still be strongly temperature-dependent without affecting the oscillation period significantly because their control coefficients are of small amplitude. As reported in [25], the Goodwin model for the circadian clock also achieves temperature compensation via this mechanism. The insulation mechanism was frequently observed in our findings. As illustrated in panels a_1_, a_2_ and c_2_ in Fig 4, only one or two reaction rates have *C*_*i*_*s* of relatively large amplitude. The insulation mechanism is particularly apparent in Fig 4*a*_1_ and 4*a*_2_, where no substantially positive control coefficients exist. These results confirm that the activation energies corresponding to the large-amplitude *C*_*i*_ are relatively small.

**Fig 5:**
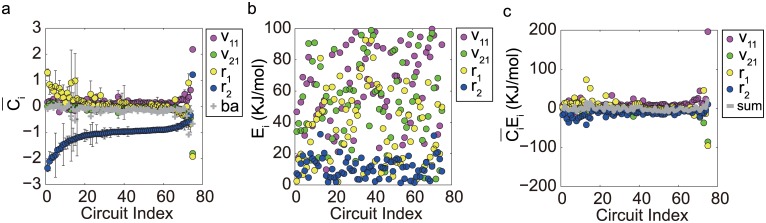
Achieving TCOs in motif C. (a) Averaged control coefficient ${\bar{C}}_{i}$ over the examined temperature range for different parameter combinations. The error bar is the standard deviation. The horizontal axis is the index for circuits with different TCO parameters for motif C. The decay rate constant *r*_2_ has the largest amplitude and dominates the oscillation period. The data were obtained by computations of 100,000 parameter samplings. (b) The activation energies for the rate constants corresponding to the control coefficients in (a). The activation energies for *r*_2_ fall primarily on the bottom. (c) is the product $\bar{C_{i}}E_{i}$ of the data in (a) and (b).

Fig 5 shows that the temperature insulation scheme was widely adopted in TCOs in motif C. The vertical axis in Fig 5*a* is the control coefficient *C*_*i*_ averaged over the examined temperature range in different TCO realizations for motif C. On average, all rate constants but one have control coefficients close to zero (refer to the blue circles for r_2_, which is the degradation rate constant for Node 2), showing that changes in most rate constants do not affect the oscillation period significantly. The unique rate constant r_2_ that has a large absolute value of *C*_*i*_ dominates the oscillation period. The activation energies depicted in Fig 5b indicate that the activation energy is lowest for *r*_2_ (blue circles). Thus, the most dangerous changes in *r*_2_ are shielded from temperature fluctuations. Fig 5c, which was generated by multiplying $\bar{C_{i}}$ in Fig 5*a* and the corresponding *E*_*i*_ in Fig 5*b*, shows that the products $\bar{C_{i}}E_{i}$ are apparently distributed in the neighborhood of the horizontal axis and that their sum is effectively zero (grey circles). In S7 and S8 Figs, more examples of temperature insulation are depicted for motifs A, B, and D as well as for several more complex topologies hybridized from the core motifs. The key for achieving TCOs in these topologies is that the oscillation period is controlled by the rate constants that are robust against temperature changes and is insensitive to other rate constants that might depend significantly on temperature. A more detailed examination of the time series for the temperature insulation mechanism in motif C is illustrated in S9 Fig and explained in the S1 Text.

**Fig 6:**
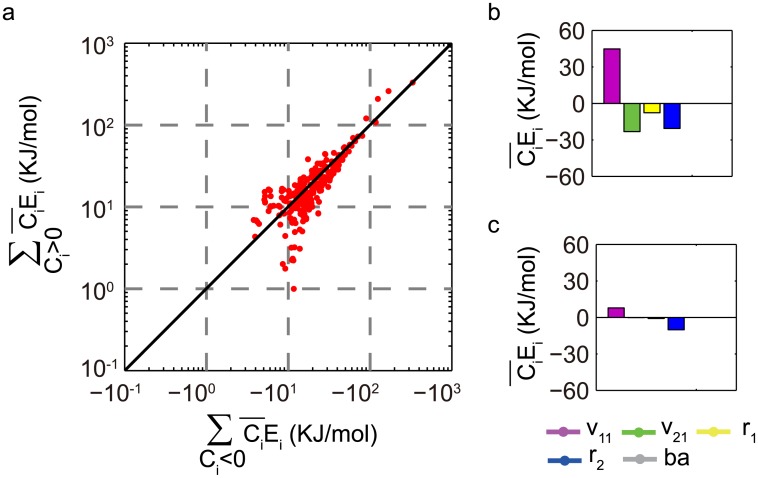
Mechanism of temperature compensation. (a) Accumulation of *C*_*i*_*E*_*i*_ with *C*_*i*_ > 0 plotted against that with *C*_*i*_ < 0 for 398 different TCO realizations in the 35 best TCO topologies. The sums $\sum_{C_{i}>0}C_{i}E_{i}$ and $\sum_{C_{i}<0}C_{i}E_{i}$ are averaged over the examined temperature range and plotted in logarithmic scale with an inverted horizontal axis. (b) The mean *C*_*i*_*E*_*i*_*s* corresponding to the *C*_*i*_~*T* dependencies in Fig 4c_1_. (c) The mean *C*_*i*_*E*_*i*_*s* corresponding to the *C*_*i*_~*T* dependencies in Fig 4c_2_. The averages were calculated over the temperature range [283K, 303K].

Fig 6 depicts the accumulated positive *C*_*i*_*E*_*i*_ against the negative *C*_*i*_*E*_*i*_ for 398 different TCO realizations in the 35 best TCO topologies. The scattering points are distributed mostly along the diagonal line, suggesting that an antagonistic balance scheme is adopted in these TCOs. For the worst TCO topologies that support oscillations but are limited in their ability to achieve a temperature-independent oscillation period, the scattering points in the $\sum_{C_{i}>0}C_{i}E_{i}vs.\sum_{C_{i}<0}C_{i}E_{i}$ plane deviate drastically from the diagonal line, indicating the violation of Eq 1 for these oscillations with a temperature-dependent period (Refer to S4 Fig). For the two distinctive temperature dependences of *C*_*i*_ in Fig 4c_1_ and 4c_2_, we further examined the mean $\bar{C_{i}}E_{i}$ contributed by the component constants. As illustrated in Fig 6b, $\bar{C_{i}}E_{i}$ for the constant *v*_11_ is a relatively large positive value, and CiEiRT2≈0. It is balanced by the negative contributions from the remaining constants, v_21_, r_1_, and r_2_. For the case of Figs 4c_2_ and 6c indicates that the *C*_*i*_*E*_*i*_ for each constant is very small and $\frac{C_{i}E_{i}}{RT^{2}}$ is effectively near zero, so that Eq 1 is satisfied by having each $\frac{C_{i}E_{i}}{RT^{2}}\approx 0$. A near-zero value of each $\frac{C_{i}E_{i}}{RT^{2}}$ is featured in the TCOs that are realized by the temperature insulation approach.

### TCO motifs as the backbone of circadian clocks across species

In the classic view of a circadian clock, the daily cellular rhythm is heavily based on a core transcription-translation feedback loop in which positive factors activate the expression of clock genes that encode negative regulators that inhibit the activities of the positive elements [3, 4]. The transcription-dependent clock mechanism, which is topologically a delayed negative feedback motif for oscillations (motif A in Fig 2a), is highly conserved across species. Table 1 demonstrates the core interactions in circadian clocks across several species, together with the corresponding simple TCO motifs or motif compositions. The circadian clocks in *Neurospora* [44], *Arabidopsis* [45, 46], *Drosophila* [47, 48], birds [49] and mammals [50, 51] share TCO motif A as their backbone, although the components vary among species. For example, in mammals, the transcription activators CLOCK and BMAL1 form complexes that bind to the conserved E/E’-box sequences in the promoter regions of the target genes *Per* and *Cry* to activate their transcription; in turn, the PER and CRY proteins form heterodimers in the cytoplasm and translocate back to the nucleus to inhibit BMAL1:CLOCK-mediated gene expression [50]. In mammals (and similarly in *Drosophila* and birds), interlocked with the core transcription feedback loop is a second transcription-translation feedback that is generated by the BMAL1:CLOCK-activated transcription of *Rev*-*Erbα* and *Rora* and subsequent repression (mediated by REV-ERB*α*) and activation (mediated by RORa) of *Bmal1*. This second feedback is topologically expressed as motif C in Table 1, where the positive loop is abbreviated by self-activation of BMAL1/CLOCK. For the mammalian circadian clock, it was revealed recently that the complex transcriptional-translational clock machinery can be reduced minimally to a simple circuit that is a composite of two oscillatory motifs, i.e., a delayed feedback loop (motif A) and repressilator (motif B) [51]. The minimal regulatory network of the mammalian circadian clock consists of three regulatory DNA elements, the E/E‘-box, the D-box in the regulatory region of *Cry1*, and the RREs in its intron, which form the delayed negative feedback loop “E/E‘-box→D-box→RRE⊸E/E‘-box” and the repressilator “E/E‘-box⊸RRE⊸D-box⊸E/E‘-box”. These two oscillatory network motifs constitute a combinational TCO topology of AB type (Table 1). In *Arabidopsis*, a repressilator structure was recently identified as a core integrated element of the complex machinery of the circadian clock [45]. The cyanobacterium circadian clock is a unique transcription-independent oscillator (motif C) whose components KaiA, KaiB and KaiC have been demonstrated to compose a temperature-compensated circadian clock in the presence of ATP *in vitro* [52].

**Table 1:** Core interactions in circadian clocks across species.

Organisms	Core circuits of positive/negative elements	TCO Motifs	Ref.
*Cyanobacteria*	↻ KaiC(P) → KaiB ⊸ KaiC(P)	C	[52]
*Neurospora*	WCC → *Frq* → FRQ ⊸ WCC	A	[44]
*Arabidopsis*	CCA1/LHY ⊸ EC ⊸ PRRs ⊸ CCA1/LHY	B	[45, 46]
	CCA1/LHY → PRRs ⊸ LHY → CCA1/LHY	A	
*Drosophila*	dCLK/CYC → *Per*/*Tim* → PER/TIM ⊸ dCLK/CYC	A	[47, 48]
	↻ CLK/CYC → *Per*/*Tim* ⊸ CLK/CYC	C	
*Avian*	CLOCK/BMAL1 → *Per*/*Cry* → PER/CRY ⊸ CLOCK/BMAL1	A	[49]
	↻CLOCK/BMAL1 → *Per*/*Cry* ⊸ CLOCK/BMAL1	C	
*Mammals*	CLOCK/BMAL1 → *Per/Cry* → PER/CRY ⊸ CLOCK/BMAL1	A	[50, 51]
	↻ CLOCK/BMAL1 → *Rev*-*Erb* ⊸ CLOCK/BMAL1	C	
	E/E‘-box → D-box → RRE ⊸ E/E‘-box	A	
	E/E‘-box ⊸ RRE ⊸ D-box ⊸ E/E‘-box	B	

The central structures and corresponding TCO motifs or motif compositions of circadian clocks from low to high organisms. X → Y (X ⊸ Y) denotes that X has a positive (negative) influence on the abundance of Y. ↻ Kai is an abbreviation for the auto-phosphorylation of KaiC with the help of KaiA. For *Drosophila*, avian and mammals, the regulation ↻ X is a reduced description of the process that X up-regulates itself by promoting the transcription of its activator. E/E‘-box, D-box, and RRE refer to the three regulatory DNA elements in the mammalian circadian clock.

## Discussion

From the general condition for TCOs (Eq 1), the temperature-independent oscillation period involves two key factors, the control coefficient Ci and the activation energy Ei. The value of Ei is locally determined by the properties of the chemical reaction steps, which depend on the specific protein structures. Mutations can alter the chemical properties of proteins and thus the values of Ei, and appropriate protein mutations would lead to satisfaction of Eq 1[21]. In this paper, we focused on the role of the other key factor, i.e., the control coefficient Ci in Eq 1. In contrast to the factor Ei, which is local, the control coefficient depends on the whole network topologies of the underlying biochemical interactions. This dependence is complex and difficult to resolve analytically. The main hypothesis of this work is that the network architecture plays an important role in TCOs. To test this hypothesis, networks for topologies preferred by TCOs were enumerated in the present study. Second, the temperature enters our models in the form of Arrhenius law. This is a simplification and assumption of the influence of temperature on gene expression. The real situation would be much more complex because gene transcription, mRNA processing, translation, protein stability, and protein-protein interactions [53] all depend strongly on temperature.

Temperature compensation is a quality that depends compositely on the oscillations, and the circadian clock is a paradigm of bi-functional machinery. Using simple gene regulatory models, we have explored theoretically the design principles and core network topologies that this ubiquitous bi-function would prefer. By enumerating all topologies with three genes with transcriptional interactions, we find four simplest network motifs for TCO: delayed negative feedback (motif A), repressilator (motif B), activator-inhibitor (motif C) and substrate-depletion (motif D). These simple network motifs are core topologies for composite oscillations to constitute the vast majority of network topologies that can achieve TCOs. The four TCO motifs fall in two categories and are biased in their capacities for oscillation and the temperature-independent period. The delayed negative feedback oscillator and repressilator are biased toward oscillations, whereas the activator-inhibitor oscillator and substrate-depletion oscillator are superior in thermal robust oscillations. Our results propose that thermally robust oscillations can be plausibly achieved by hybridizing these two categories of network motifs.

Temperature compensation was observed to be accomplished via two mechanisms. The first is the distributed scenario or antagonistic balance, which imposes global constraints on all parameters. The oscillation period sensitively depends on an extensive number of parameters, and period-increasing effects are delicately cancelled out by the opposing period-decreasing effects to achieve a temperature-independent oscillation period. The second approach identified by our findings is the temperature insulation mechanism, in which the oscillation period is determined by a very few temperature-independent or only slightly temperature-dependent parameters, although other parameters could still strongly depend on the temperature. Apparently, temperature compensation based on the distributed mechanism relies on all parameters and is fragile against perturbations such as gene mutations. The insulation scheme is superior to the distributed mechanism because it is more robust against parameter variations. Indeed, as summarized in [22], the robustness against extensive gene mutations of circadian clocks has been experimentally verified in *N*. *crassa* and *D*. *melanogaster*. The mechanism in which the oscillation period is controlled by a few temperature-insensitive factors has been theoretically investigated in the cyanobacterial circadian clock [54]. In this instance, the ATPase-mediated delay dominates the oscillation period, and a thermally robust clock is ensured by the insensitivity of ATPase to temperature variations. Our results indicate that the oscillation amplitude of TCOs depends on the temperature. Beyond the temperature-independent oscillation period that we focused on, temperature entrainment is another defining property of circadian clocks that was not discussed in this work. In a circadian clock, the oscillation phase can be shifted by temperature pulses, and the oscillation period can also be entrained by temperature oscillations of small amplitude [55]. In addition, the cycle shape and the phase relationship can remain unchanged at different but constant temperatures [26]. These properties might cast additional constraints on the topology structures for circadian clocks.

## Methods

### Enumeration of 2-node and 3-node networks

We adopted a 3a adjacency matrix *J* to describe the topological structure of the networks. The element *J*_*ij*_ can be 1, -1 or 0, which indicate that Node *j* activates Node *i*, inhibits Node *i*, or has no interaction with Node *i*, respectively. There are nine possible regulations among the three nodes, and each regulation might be positive, negative or null. This yields a total of 3^9^ = 19683 possible networks. Those networks with an isolated node with no regulation of the remaining nodes were considered 2-node networks. To reduce the network space for exhaustive exploration, we excluded redundant networks that are topologically equivalent (e.g., the topology A ⊸ B ⊸ C ⊸ A is topologically equivalent to the one in which B ⊸ A ⊸ C ⊸ B.). We thus have a total of 2423 distinct topologies, of which there are 2384 three-node topologies and 39 two-node topologies

### Ordinary differential equations for genetic regulatory networks

To describe dynamical behaviors of genetic interaction networks, we adopted the general model for transcription interactions [37]. The protein production rate of gene *i* was determined by the matrix elements *J*_*ij*_, *j* = 1,2,3, whose values represent the nature of regulation. For the case in which a gene is regulated by more than one transcription factor, competitive binding at the regulatory site was considered. The rate contribution of transcriptional regulation was given by a multi-dimensional input function of Hill-function form [37]. Together with the basal expression and a linear form of the degradation rate, the full protein production rate for gene *i* takes the following form;

$$\frac{dx_{i}}{dt}=\delta_{i}+\frac{\sum_{\left(j|J_{ij}=1\right)}v_{ij}{\left(\frac{x_{j}}{K_{ij}}\right)}^{n}}{1+\sum_{\left(j|J_{ij}\neq 0\right)}{\left(\frac{x_{j}}{K_{ij}}\right)}^{n}}-r_{i}x_{i}$$ (2)

where *x*_*i*_ is the protein concentration expressed from gene *i*; *K*_*ij*_ is the dissociation constant; and *n* is the Hill coefficient. We fix *n* = 3; a higher value of *n* would promote oscillation but would not significantly alter our results. *δ*_*i*_ is the basal rate (*δ*_*i*_ = 0.01). The maximal rate *v*_*ij*_ and the decay coefficient *r*_*i*_ are assumed to depend on temperature. The rate of chemical reactions has generally a strong temperature dependence. Because most biochemical reactions have an energy barrier that is overcome with the help of enzymes, the rate could be plausibly written in the Arrhenius form:

$$k=Ae^{-\frac{E}{RT}},$$ (3)

where *E* is the activation energy and *A*, *R* and *T* are the pre-exponential factor, the gas constant and the Kelvin temperature, respectively. Considering that the basal expression level *δ*_*i*_ is rather small compared to the other rates and that the dissociation constant *K*_*ij*_ is the ratio of the dissociation to association rate, we assumed that the parameters *δ*_*i*_ and *K*_*ij*_ were fixed in our model. Protein synthetic processes depend on temperature in a complex manner [38, 39]. The temperature dependence given by the Arrhenius equation in our model provides theoretically a simplified estimate of the complicated temperature dependence of actual protein synthesis. The parameters of *K*_*ij*_, *v*_*ij*_ and *r*_*i*_ were sampled uniformly at the logarithmic scale using the Latin hypercube sampling method. The sampling ranges of these parameters were *K*_*ij*_~10^−2^ − 10^2^ a.u., *v*_*ij*_~10^0^ − 10^2^ a.u. and *r*_*i*_~10^−1^ − 10^1^ a.u. In addition, the parameters of *E*_*i*_ were sampled uniformly from 1KJ/mol to 100 KJ/mol.

### Assessment of TCO capacity for each oscillatory circuit

For a given network topology and a randomly sampled set of parameters, we first examined whether spontaneous oscillations are sustained under proper initial conditions. For a circuit that oscillates in the whole range of temperature [283K, 303K], we verified the oscillation periods at five temperatures uniformly spaced in the temperature range (i.e., 283K, 288K, 293K, 298K, 303K). The capacity for an oscillatory circuit to achieve TCO was estimated by the relative standard deviation (RSD) of the oscillation periods. For an RSD less than 10%, the circuit was considered capable of performing compensated oscillations.

### Calculation for the control coefficients of the oscillation period

The control coefficient of the oscillation period (*C*_*i*_) is defined as ∂*lnp/*∂*lnk*_*i*_ and is numerically calculated. For each 1% increment in each rate constant, we calculate the corresponding relative change in the oscillation period. The control coefficient (*C*_*i*_) is obtained by calculating the ratio ΔPPΔkiki.

## Supporting Information

S1 Table The values of Q, q and q/Q for simple motifs in 100,000 randomly searched parameter sets. (DOCX)

S1 Fig Distribution of oscillatory topologies in q-value space. The number of topologies falls off exponentially with increasing q-value, with very few topologies having large q-values. A total of 1504 oscillatory topologies were obtained by checking all possible networks and randomly sampling 10,000 parameter combinations. The capacity for temperature compensation was evaluated by the number (i.e., q-value) of parameter samplings that can achieve roughly fixed oscillation periods.
(TIF)

S2 Fig The simplest combinational networks of BC and AC. There are one kind of BC combination and three kinds of AC combinations. The numbers under each network represent the corresponding Q-value and q-value in 10,000 sets of sampling. Different AC combinations have different effects in promoting TCOs. The last two kinds of AC combinations are much better than the first one in achieving TCOs.
(EPS)

S3 Fig The networks in the uppermost row in Fig 2b. There are two kinds of ABC combinations, five kinds of AC combinations, one kind of BC combination and three kinds of topologies only containing motif C. The numbers under each network represent the corresponding Q-value and q-value in 10,000 sets of sampling.
(EPS)

S4 FigOscillations with a temperature-dependent period.The sum $\sum_{C_{i}<0}C_{i}E_{i}$ is plotted against $\sum_{C_{i}<0}C_{i}E_{i}$ for non-TCO oscillations generated by 400 circuits with the worst 35 TCO topologies. The sums $\sum_{C_{i}<0}C_{i}E_{i}$ and $\sum_{C_{i}<0}C_{i}E_{i}$ were averaged over the evaluated temperature range and plotted on a logarithmic scale with an inverted horizontal axis. The scattering points deviate drastically from the diagonal line. These non-TCO circuits are randomly generated by the worst TCO networks. The *C*_*i*_*E*_*i*_*s* are predominantly negative; thus, an increase in the temperature accelerates the oscillations and decreases the period.(TIF)

S5 Fig Temperature-dependence of control coefficients multiplied by activation energies for simple motifs. The temperature dependence of elasticity *C*_*i*_ is demonstrated for motifs A (*a*_1_, *a*_2_), B (*b*_1_, *b*_2_), C (*c*_1_, *c*_2_) and D (*d*_1_, *d*_2_). Each topology has one set of TCO parameters that can achieve antagonistic balance (*a*_1_, *b*_1,_ *c*_1_, *d*_1_ in the upper row) and another non-TCO set that is not balanced (*a*_2_, *b*_2,_ *c*_2_, *d*_2_ in the lower row).
(TIF)

S6 Fig Temperature dependence of the control coefficients multiplied by the activation energies for combinational topologies. Examples of *C*_*i*_*E*_*i*_ for a topology composed of motifs A and C (*a*_1_, *a*_2_) and a topology of motifs B and C (*b*_1_, *b*_2_), each with one set of TCO (up row) and non-TCO (low row) parameters.
(TIF)

S7 Fig The mean and deviation of the control coefficients for motifs A, B and D. The sensitivity *C*_*i*_ is averaged over the temperature range from 283K to 303K. The error bar is the standard deviation of elasticity *C*_*i*_. The horizontal axis is the index for circuits with different parameters but a common topology. The data were obtained by expanding the sampling from 10,000 to 100,000 to examine more TCO circuits.
(TIF)

S8 Fig The mean and deviation of the control coefficients for combinational topologies. The temperature-averaged control coefficients for combinational topologies of motifs A and C (a), B and C (b), A and D (c), C and C (d), A, B and C (e), and A, C and D (f). In the data for these TCO topologies, there is normally a dominant parameter with a relatively larger control coefficient that can exert a strong influence on the oscillation frequency.
(TIF)

S9 Fig Temporal division of time-series for motif C. The oscillation period is divided roughly into four stages according to the concentration of node 2: the mainly rising phase *τ*_1_, the mainly falling phase *τ*_3_ and the transient phases between the rising and falling phases *τ*_2_,*τ*_4_. The oscillation period is mainly determined by the rising (*τ*_1_) and falling (*τ*_3_) phases, which are dominated by the parameter *r*_2_
(TIF)

S1 Text Supporting Information, contains Temperature sensitivity of the oscillation period for TCO topologies, the TCO mechanism for motif C and motif B, parameters for circuits depicted in Figs 4 and 6. (DOCX)

## References

[1] Oleksiuk O, Jakovljevic V, Vladimirov N, Carvalho R, Paster E, Ryu WS, et al Thermal robustness of signaling in bacterial chemotaxis. Cell. 2011;145(2):312–21. 10.1016/j.cell.2011.03.013 21496648 PMC3098529

[2] Shimizu H, Woodcock SA, Wilkin MB, Trubenová B, Monk NA, Baron M. Compensatory flux changes within an endocytic trafficking network maintain thermal robustness of Notch signaling. Cell. 2014;157(5):1160–74. 10.1016/j.cell.2014.03.050 24855951 PMC4032575

[3] Bell-Pedersen D, Cassone VM, Earnest DJ, Golden SS, Hardin PE, Thomas TL, et al Circadian rhythms from multiple oscillators: lessons from diverse organisms. Nat Rev Genet. 2005;6(7):544–56. 10.1038/nrg1633 15951747 PMC2735866

[4] Eckel-Mahan K, Sassone-Corsi P. Metabolism and the circadian clock converge. Physiol Rev. 2013;93(1):107–35. 10.1152/physrev.00016.2012 23303907 PMC3781773

[5] Denlinger DL, Giebultowicz J, Saunders DS. Insect timing: circadian rhythmicity to seasonality: Elsevier; 2001.

[6] Dunlap JC. Molecular bases for circadian clocks. Cell. 1999;96(2):271–90. 9988221 10.1016/s0092-8674(00)80566-8

[7] Harmer SL, Panda S, Kay SA. Molecular bases of circadian rhythms. Annu Rev Cell Dev Biol. 2001;17(1):215–53. 11687489 10.1146/annurev.cellbio.17.1.215

[8] Hastings JW, Sweeney BM. On the mechanism of temperature independence in a biological clock. Proc Natl Acad Sci U S A. 1957;43(9):804–11. 16590089 10.1073/pnas.43.9.804 PMC534330

[9] Pittendrigh CS. On temperature independence in the clock system controlling emergence time in Drosophila. Proc Natl Acad Sci U S A. 1954;40(10):1018–29. 16589583 10.1073/pnas.40.10.1018 PMC534216

[10] Gould PD, Locke JC, Larue C, Southern MM, Davis SJ, Hanano S, et al The molecular basis of temperature compensation in the Arabidopsis circadian clock. The Plant Cell. 2006;18(5):1177–87. 10.1105/tpc.105.039990 16617099 PMC1456873

[11] Ruoff P. Introducing temperature-compensation in any reaction kinetic oscillator model. J Theor Biol. 1992;23(2):92–9.

[12] Peter Ruoff LR. The temperature compensated Goodwin model simulates many circadian properties. J Theor Biol. 1996:179, 275–85.

[13] Ruoff P, Rensing L, Kommedal R, Mohsenzadeh S. Modeling temperature compensation in chemical and biological oscillators. Chronobiol Int. 1997;14(5):499–510. 9298285 10.3109/07420529709001471

[14] Hong CI, Tyson JJ. A Proposal for Temperature Compensation of the Orcadian Rhythm in Drosophila Based on Dimerization of the Per Protein. Chronobiol Int. 1997;14(5):521–9. 9298287 10.3109/07420529709001473

[15] Ruoff P, Christensen MK, Wolf J, Heinrich R. Temperature dependency and temperature compensation in a model of yeast glycolytic oscillations. Biophys Chem. 2003;106(2):179–92. 14556906 10.1016/s0301-4622(03)00191-1

[16] Ruoff P, Christensen MK, Sharma VK. PER/TIM-mediated amplification, gene dosage effects and temperature compensation in an interlocking-feedback loop model of the Drosophila circadian clock. J Theor Biol. 2005;237(1):41–57. 10.1016/j.jtbi.2005.03.030 15935389

[17] Kurosawa G, Iwasa Y. Temperature compensation in circadian clock models. J Theor Biol. 2005;233(4):453–68. 10.1016/j.jtbi.2004.10.012 15748908

[18] Ruoff P, Zakhartsev M, Westerhoff HV. Temperature compensation through systems biology. FEBS J. 2007;274(4):940–50. 10.1111/j.1742-4658.2007.05641.x 17227386

[19] Takeuchi T, Hinohara T, Kurosawa G, Uchida K. A temperature-compensated model for circadian rhythms that can be entrained by temperature cycles. J Theor Biol. 2007;246(1):195–204. 10.1016/j.jtbi.2006.12.028 17275853

[20] Bodenstein C, Heiland I, Schuster S. Calculating activation energies for temperature compensation in circadian rhythms. Phys Biol. 2011;8(5):056007 10.1088/1478-3975/8/5/056007 21891835

[21] Hussain F, Gupta C, Hirning AJ, Ott W, Matthews KS, Josić K, et al Engineered temperature compensation in a synthetic genetic clock. Proc Natl Acad Sci U S A. 2014;111(3):972–7. 10.1073/pnas.1316298111 24395809 PMC3903251

[22] Hong CI, Conrad ED, Tyson JJ. A proposal for robust temperature compensation of circadian rhythms. Proc Natl Acad Sci U S A. 2007;104(4):1195–200. 10.1073/pnas.0601378104 17229851 PMC1773060

[23] van Zon JS, Lubensky DK, Altena PR, ten Wolde PR. An allosteric model of circadian KaiC phosphorylation. Proc Natl Acad Sci U S A. 2007;104(18):7420–5. 10.1073/pnas.0608665104 17460047 PMC1863508

[24] Hatakeyama TS, Kaneko K. Generic temperature compensation of biological clocks by autonomous regulation of catalyst concentration. Proc Natl Acad Sci U S A. 2012;109(21):8109–14. 10.1073/pnas.1120711109 22566655 PMC3361444

[25] François P, Despierre N, Siggia ED. Adaptive temperature compensation in circadian oscillations. PLoS Comput Biol. 2012;8(7):e1002585 10.1371/journal.pcbi.1002585 22807663 PMC3395600

[26] Kidd PB, Young MW, Siggia ED. Temperature compensation and temperature sensation in the circadian clock. Proc Natl Acad Sci U S A. 2015;112(46):E6284–E92. 10.1073/pnas.1511215112 26578788 PMC4655526

[27] Hogenesch JB, Ueda HR. Understanding systems-level properties: timely stories from the study of clocks. Nat Rev Genet. 2011;12(6):407–16. 10.1038/nrg2972 21556016

[28] Barkal N, Leibler S. Robustness in simple biochemical networks. Nature. 1997;387(6636):913–7. 10.1038/43199 9202124

[29] Shen-Orr SS, Milo R, Mangan S, Alon U. Network motifs in the transcriptional regulation network of Escherichia coli. Nat Genet. 2002;31(1):64–8. 10.1038/ng881 11967538

[30] Wagner A. Circuit topology and the evolution of robustness in two-gene circadian oscillators. Proc Natl Acad Sci U S A. 2005;102(33):11775–80. 10.1073/pnas.0501094102 16087882 PMC1183445

[31] Lim WA, Lee CM, Tang C. Design principles of regulatory networks: searching for the molecular algorithms of the cell. Mol Cell. 2013;49(2):202–12. 10.1016/j.molcel.2012.12.020 23352241 PMC3664230

[32] Castillo-Hair SM, Villota ER, Coronado AM. Design principles for robust oscillatory behavior. Syst Synth Biol. 2015;9(3):125–33. 10.1007/s11693-015-9178-6 26279706 PMC4531878

[33] Ma W, Trusina A, El-Samad H, Lim WA, Tang C. Defining network topologies that can achieve biochemical adaptation. Cell. 2009;138(4):760–73. 10.1016/j.cell.2009.06.013 19703401 PMC3068210

[34] Shah NA, Sarkar CA. Robust network topologies for generating switch-like cellular responses. PLoS Comput Biol. 2011;7(6):e1002085 10.1371/journal.pcbi.1002085 21731481 PMC3121696

[35] Yan L, Ouyang Q, Wang H. Dose-response aligned circuits in signaling systems. PLoS One. 2012;7(4):e34727 10.1371/journal.pone.0034727 22496849 PMC3320644

[36] Cotterell J, Sharpe J. An atlas of gene regulatory networks reveals multiple three-gene mechanisms for interpreting morphogen gradients. Mol Syst Biol. 2010;6(1):425. 21045819 10.1038/msb.2010.74 PMC3010108

[37] Alon U. An introduction to systems biology: design principles of biological circuits: CRC press; 2006.

[38] Farewell A, Neidhardt FC. Effect of temperature on in vivo protein synthetic capacity in Escherichia coli. J Bacteriol. 1998;180(17):4704–10. 9721314 10.1128/jb.180.17.4704-4710.1998 PMC107486

[39] Yun HS, Hong J, Lim HC. Regulation of ribosome synthesis in Escherichia coli: Effects of temperature and dilution rate changes. Biotechnol Bioeng. 1996;52(5):615–24. 10.1002/(SICI)1097-0290(19961205)52:5<615::AID-BIT9>3.0.CO;2-M 18629935

[40] Iman RL, Davenport JM, Zeigler DK. Latin hypercube sampling (program user's guide).[LHC, in FORTRAN]. Sandia Labs., Albuquerque, NM (USA), 1980.

[41] Goodwin BC. Oscillatory behavior in enzymatic control processes. Adv Enzyme Regul. 1965;3:425–37. 5861813 10.1016/0065-2571(65)90067-1

[42] Elowitz MB, Leibler S. A synthetic oscillatory network of transcriptional regulators. Nature. 2000;403(6767):335–8. 10.1038/35002125 10659856

[43] Marland E, Keizer J. Computational Cell Biology. Fall CP. 2002.

[44] Dunlap JC, Loros JJ. How fungi keep time: circadian system in Neurospora and other fungi. Curr Opin Microbiol. 2006;9(6):579–87. 10.1016/j.mib.2006.10.008 17064954

[45] Pokhilko A, Fernández AP, Edwards KD, Southern MM, Halliday KJ, Millar AJ. The clock gene circuit in Arabidopsis includes a repressilator with additional feedback loops. Mol Syst Biol. 2012;8(1):574. 22395476 10.1038/msb.2012.6 PMC3321525

[46] Imaizumi T. Arabidopsis circadian clock and photoperiodism: time to think about location. Curr Opin Plant Biol. 2010;13(1):83–9. 10.1016/j.pbi.2009.09.007 19836294 PMC2818179

[47] Glossop NR, Lyons LC, Hardin PE. Interlocked feedback loops within the Drosophila circadian oscillator. Science. 1999;286(5440):766–8. 10531060 10.1126/science.286.5440.766

[48] Peschel N, Helfrich-Förster C. Setting the clock–by nature: circadian rhythm in the fruitfly Drosophila melanogaster. FEBS Lett. 2011;585(10):1435–42. 10.1016/j.febslet.2011.02.028 21354415

[49] Cassone VM. Avian circadian organization: a chorus of clocks. Front Neuroendocrinol. 2014;35(1):76–88. 10.1016/j.yfrne.2013.10.002 24157655 PMC3946898

[50] Partch CL, Green CB, Takahashi JS. Molecular architecture of the mammalian circadian clock. Trends Cell Biol. 2014;24(2):90–9. 10.1016/j.tcb.2013.07.002 23916625 PMC3946763

[51] Ukai-Tadenuma M, Yamada RG, Xu H, Ripperger JA, Liu AC, Ueda HR. Delay in feedback repression by cryptochrome 1 is required for circadian clock function. Cell. 2011;144(2):268–81. 10.1016/j.cell.2010.12.019 21236481

[52] McClung CR. The cyanobacterial circadian clock is based on the intrinsic ATPase activity of KaiC. Proc Natl Acad Sci U S A. 2007;104(43):16727–8. 10.1073/pnas.0708757104 17940004 PMC2040479

[53] Lahiri K, Vallone D, Gondi SB, Santoriello C, Dickmeis T, Foulkes NS. Temperature regulates transcription in the zebrafish circadian clock. PLoS Biol. 2005;3(11):e351 10.1371/journal.pbio.0030351 16176122 PMC1233578

[54] Phong C, Markson JS, Wilhoite CM, Rust MJ. Robust and tunable circadian rhythms from differentially sensitive catalytic domains. Proc Natl Acad Sci U S A. 2013;110(3):1124–9. 10.1073/pnas.1212113110 23277568 PMC3549141

[55] Bodenstein C, Heiland I, Schuster S. Temperature compensation and entrainment in circadian rhythms. Phys Biol. 2012;9(3):036011 10.1088/1478-3975/9/3/036011 22683844

