# Supplementary material for: Robust network topologies for generating oscillations with temperature-independent periods
Source: PLoS One. 2017 Feb 2;12(2):e0171263. doi: 10.1371/journal.pone.0171263 (PMC5289577; doi:10.1371/journal.pone.0171263)
Supplement: S1 Table — (DOCX) [file pone.0171263.s001.docx]

| Motif | A | B | C | D |
| --- | --- | --- | --- | --- |
| Q | 2446 | 2448 | 481 | 78 |
| q | 43 | 141 | 76 | 14 |
| q/Q | 2% | 6% | 16% | 18% |
