# Supplementary figures and images for: Robust network topologies for generating oscillations with temperature-independent periods
Source: PLoS One. 2017 Feb 2;12(2):e0171263. doi: 10.1371/journal.pone.0171263 (PMC5289577; doi:10.1371/journal.pone.0171263)

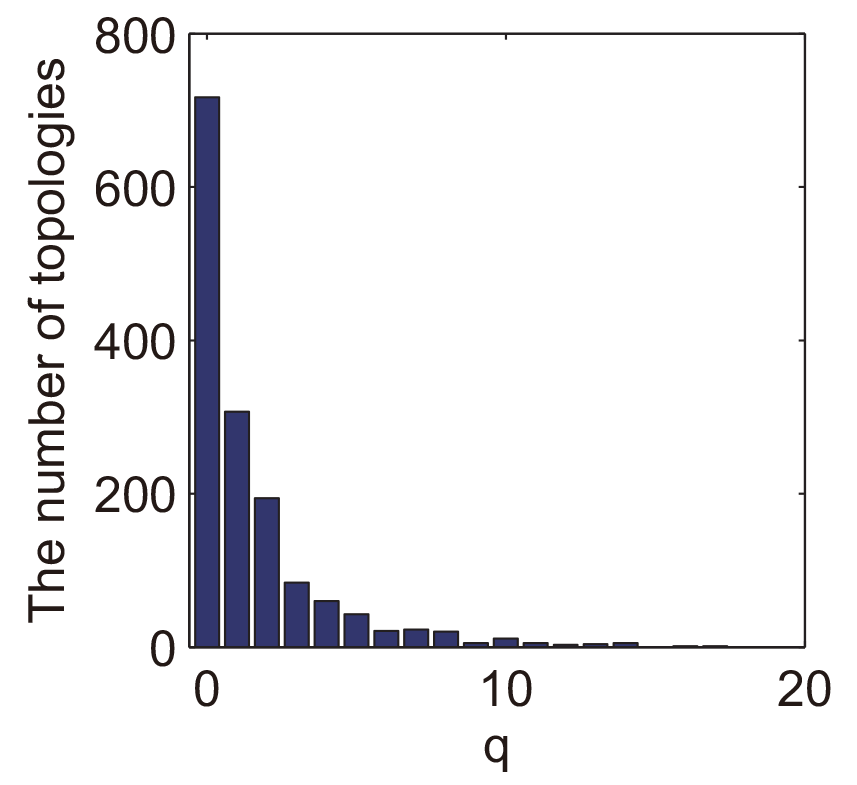

Supplement: S1 Fig — The number of topologies falls off exponentially with increasing q-value, with very few topologies having large q-values. A total of 1504 oscillatory topologies were obtained by checking all possible networks and randomly sampling 10,000 parameter combinations. The capacity for temperature compensation was evaluated by the number (i.e., q-value) of parameter samplings that can achieve roughly fixed oscillation periods. (TIF) [file pone.0171263.s002.tif]

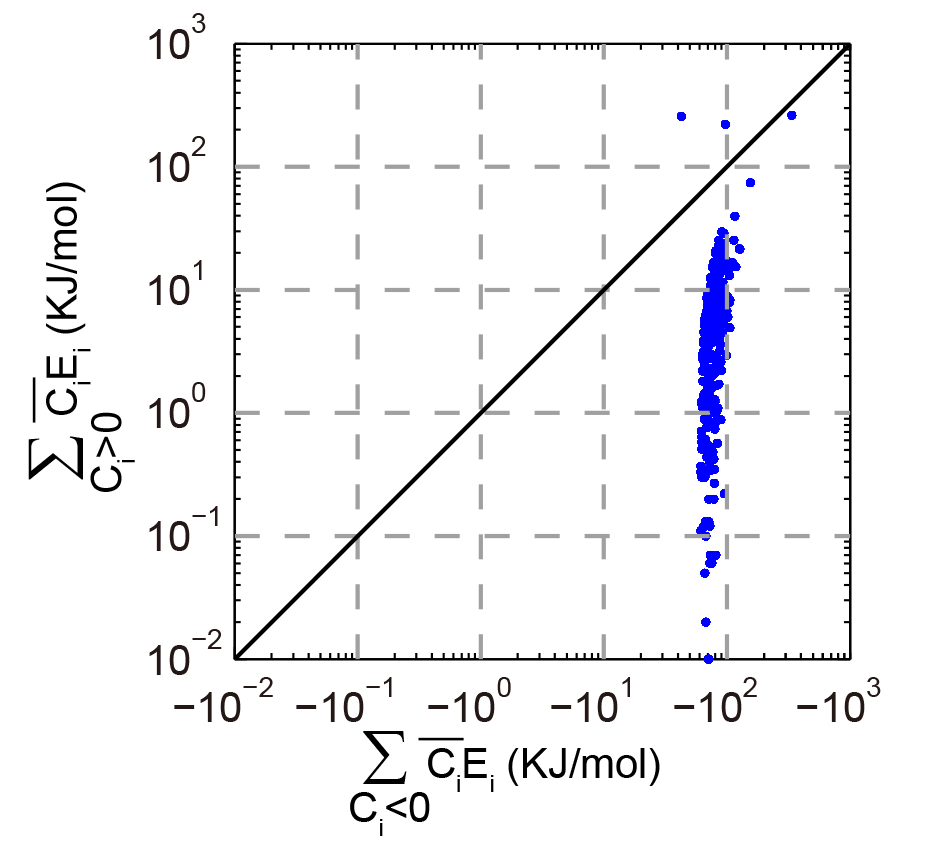

Supplement: S4 Fig — The sum ∑Ci<0CiEi is plotted against ∑Ci<0CiEi for non-TCO oscillations generated by 400 circuits with the worst 35 TCO topologies. The sums ∑Ci<0CiEi and ∑Ci<0CiEi were averaged over the evaluated temperature range and plotted on a logarithmic scale with an inverted horizontal axis. The scattering points deviate drastically from the diagonal line. These non-TCO circuits are randomly generated by the worst TCO networks. The CiEis are predominantly negative; thus, an increase in the temperature accelerates the oscillations and decreases the period. (TIF) [file pone.0171263.s005.tif]

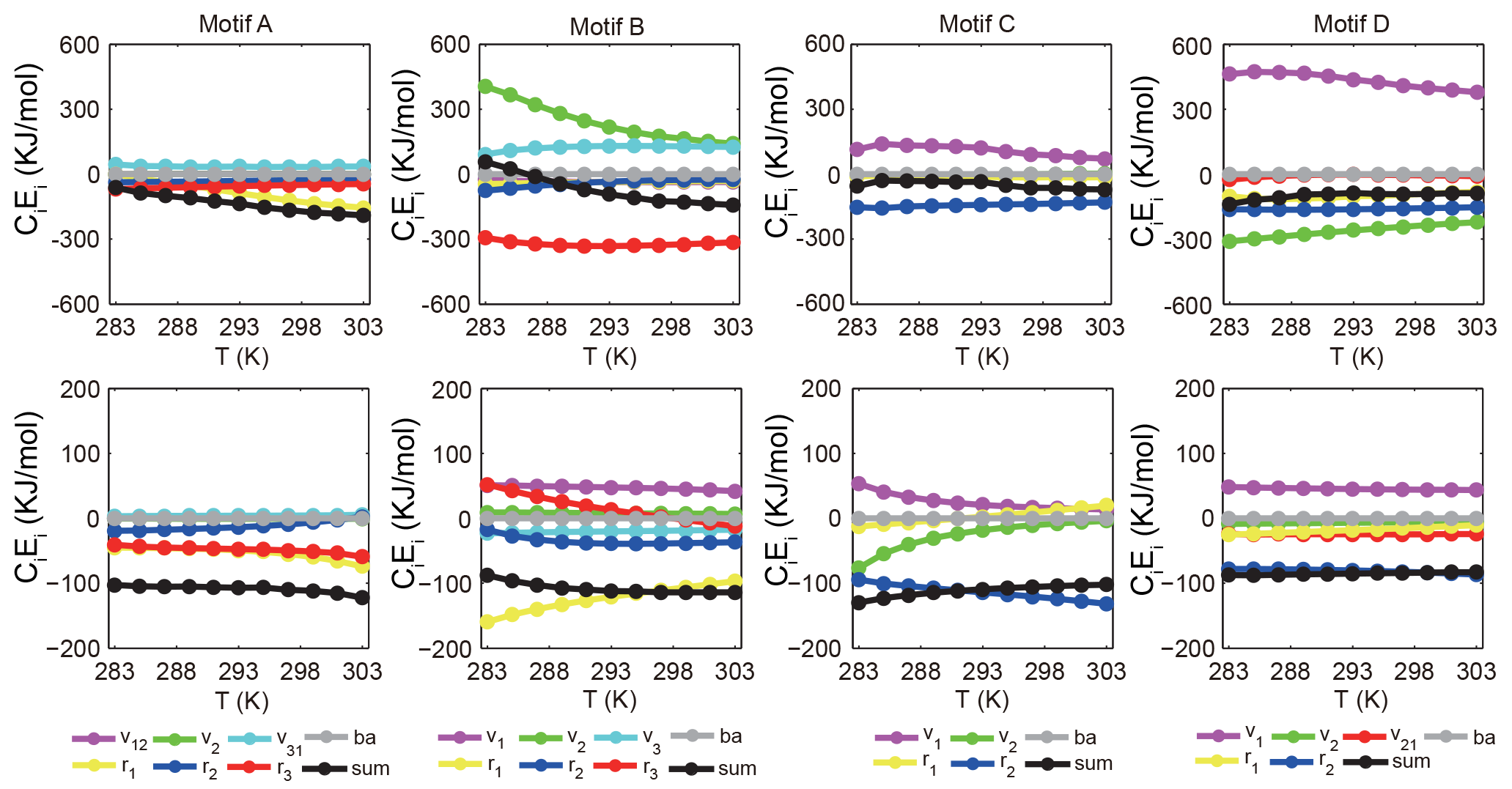

Supplement: S5 Fig — The temperature dependence of elasticity Ci is demonstrated for motifs A (a1, a2), B (b1, b2), C (c1, c2) and D (d1, d2). Each topology has one set of TCO parameters that can achieve antagonistic balance (a1, b1, c1, d1 in the upper row) and another non-TCO set that is not balanced (a2, b2, c2, d2 in the lower row). (TIF) [file pone.0171263.s006.tif]

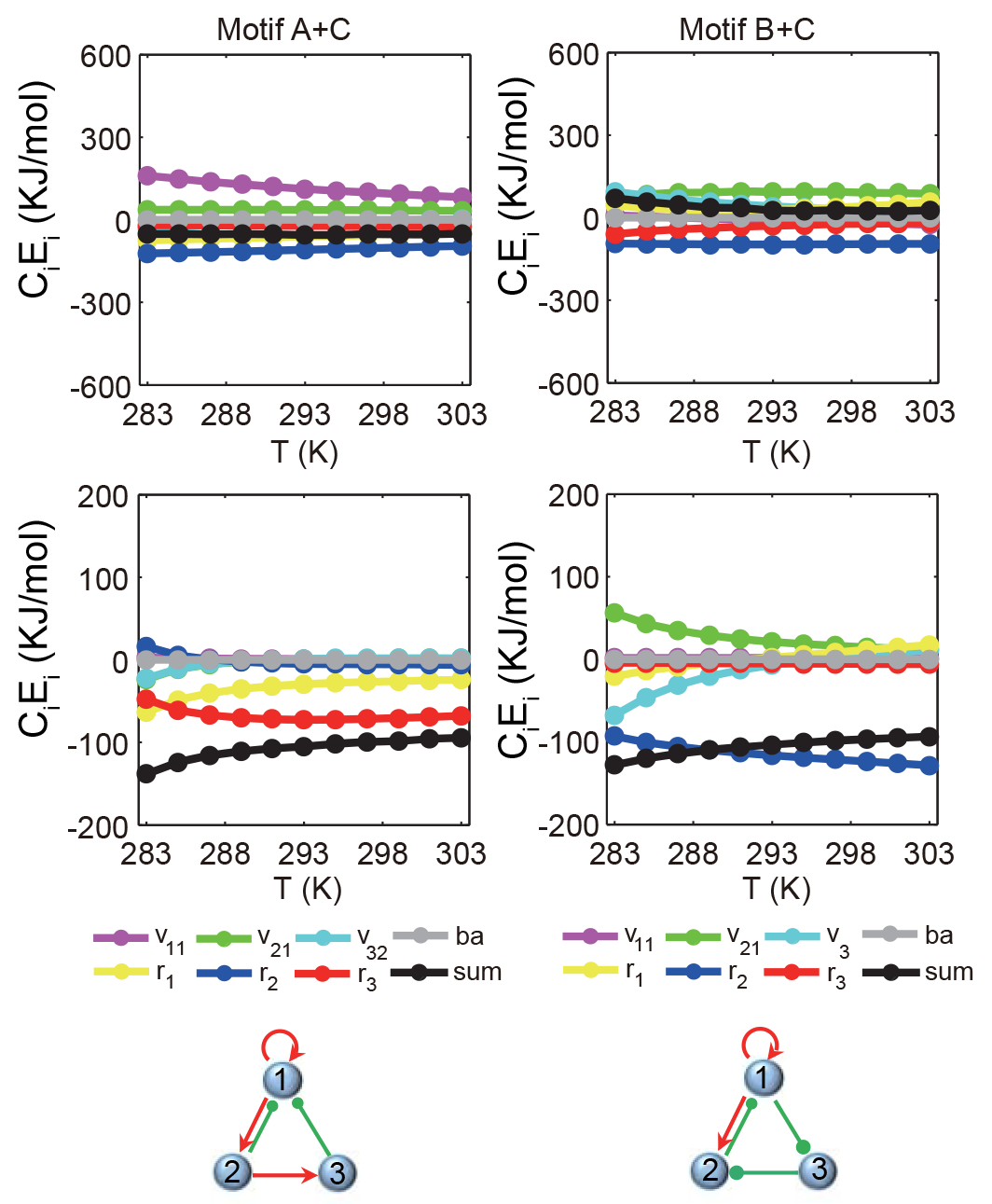

Supplement: S6 Fig — Examples of CiEi for a topology composed of motifs A and C (a1, a2) and a topology of motifs B and C (b1, b2), each with one set of TCO (up row) and non-TCO (low row) parameters. (TIF) [file pone.0171263.s007.tif]

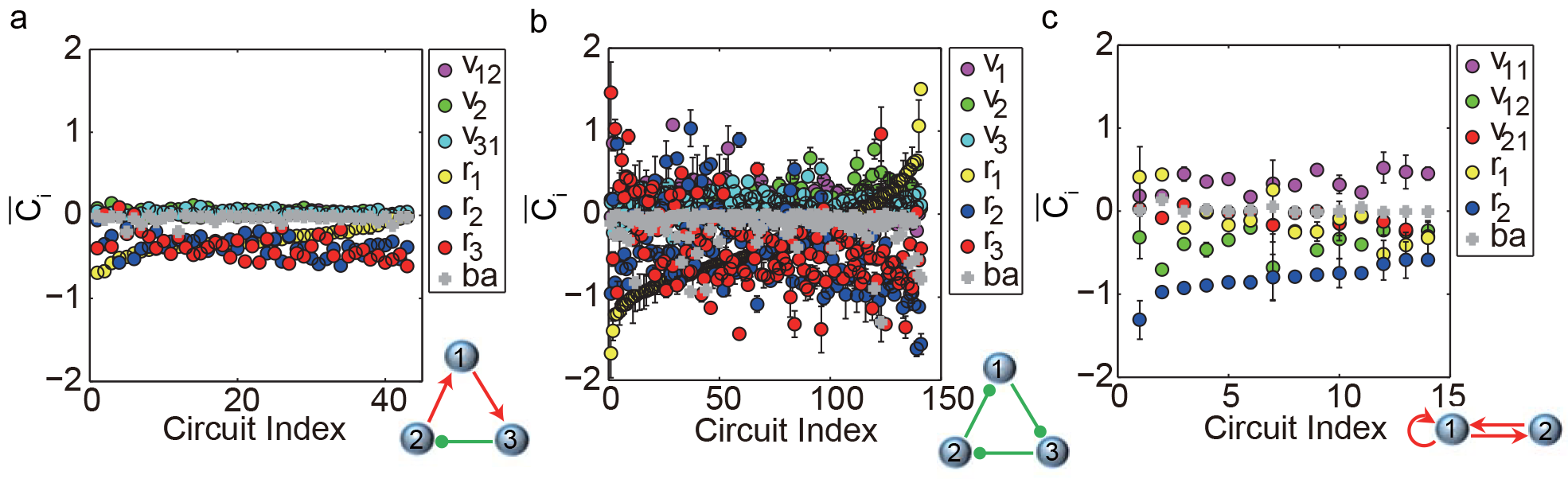

Supplement: S7 Fig — The sensitivity Ci is averaged over the temperature range from 283K to 303K. The error bar is the standard deviation of elasticity Ci. The horizontal axis is the index for circuits with different parameters but a common topology. The data were obtained by expanding the sampling from 10,000 to 100,000 to examine more TCO circuits. (TIF) [file pone.0171263.s008.tif]

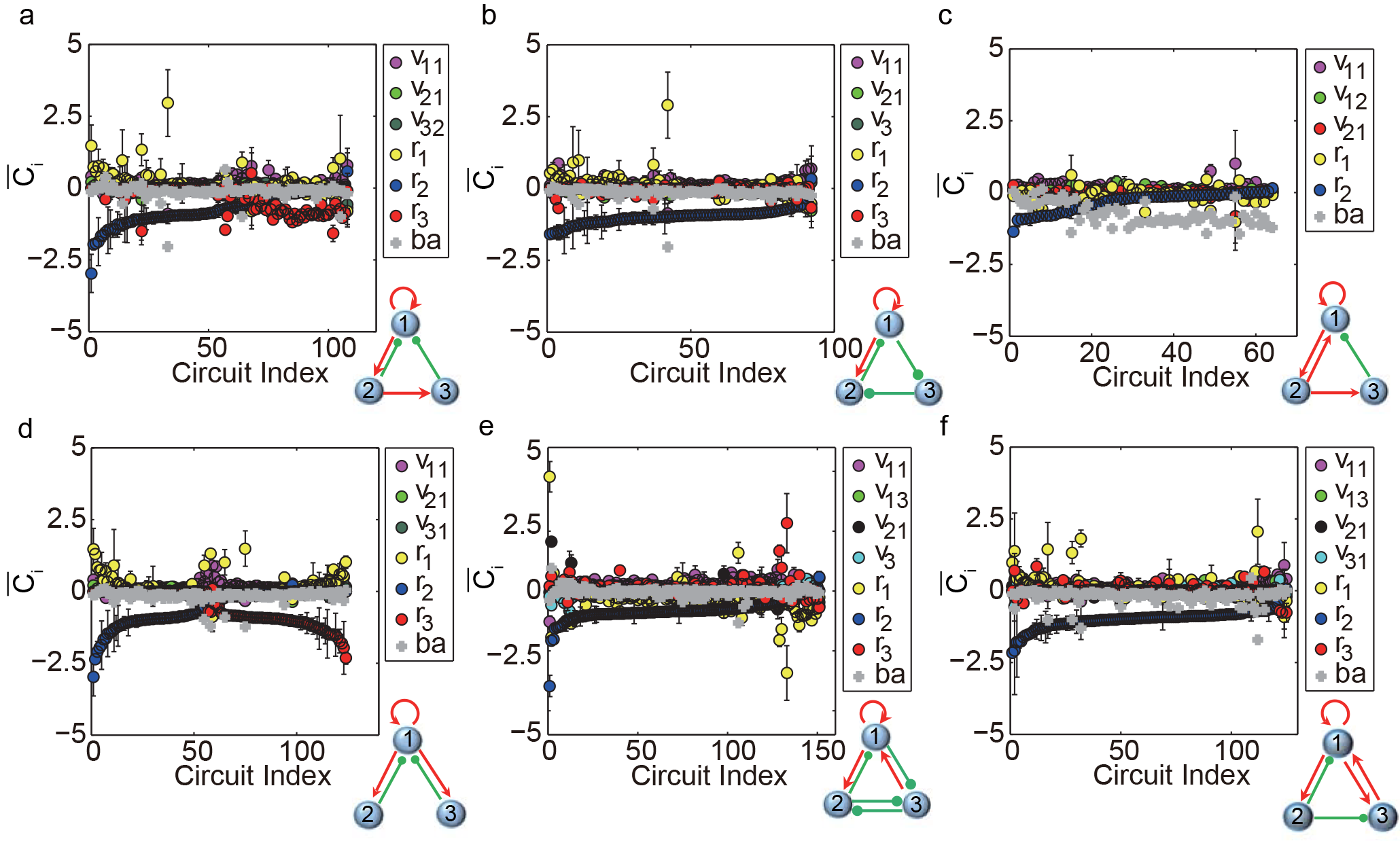

Supplement: S8 Fig — The temperature-averaged control coefficients for combinational topologies of motifs A and C (a), B and C (b), A and D (c), C and C (d), A, B and C (e), and A, C and D (f). In the data for these TCO topologies, there is normally a dominant parameter with a relatively larger control coefficient that can exert a strong influence on the oscillation frequency. (TIF) [file pone.0171263.s009.tif]

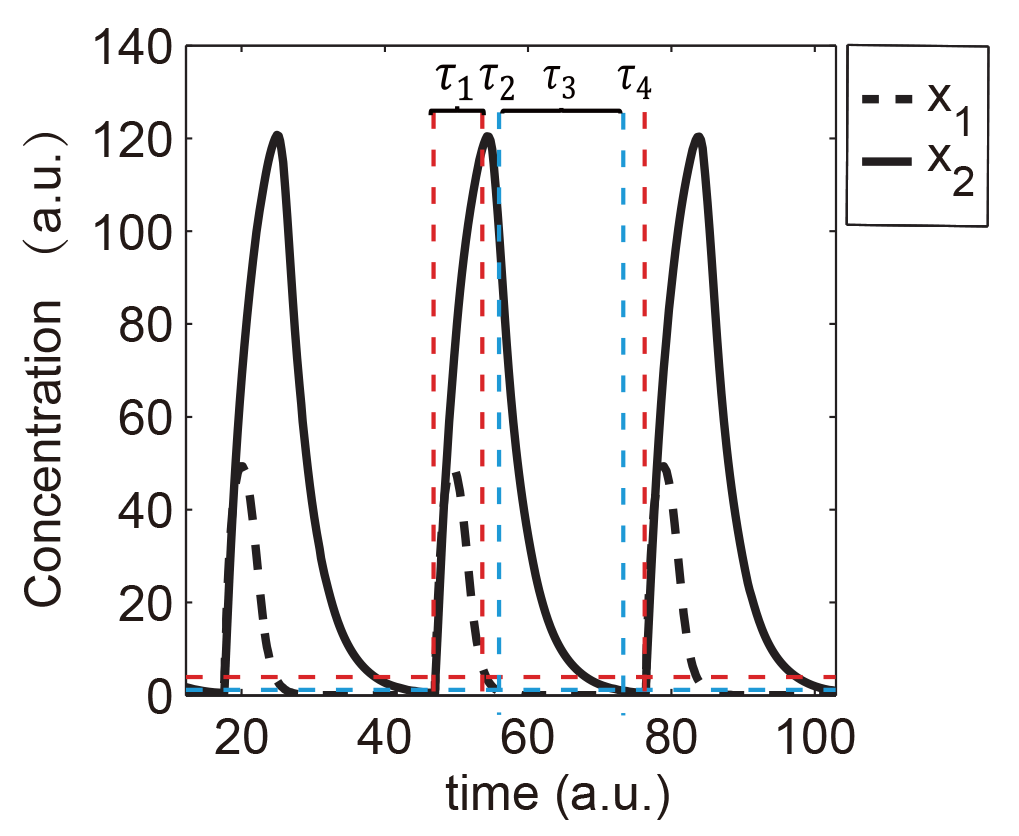

Supplement: S9 Fig — The oscillation period is divided roughly into four stages according to the concentration of node 2: the mainly rising phase τ1, the mainly falling phase τ3 and the transient phases between the rising and falling phases τ2,τ4. The oscillation period is mainly determined by the rising (τ1) and falling (τ3) phases, which are dominated by the parameter r2 (TIF) [file pone.0171263.s010.tif]
