# Supplementary material for: Robust network topologies for generating oscillations with temperature-independent periods
Source: PLoS One. 2017 Feb 2;12(2):e0171263. doi: 10.1371/journal.pone.0171263 (PMC5289577; doi:10.1371/journal.pone.0171263)
Supplement: S1 Text — (DOCX) [file pone.0171263.s011.docx]

# Supporting Information

## Temperature sensitivity of the oscillation period for TCO topologies. Based on the temperature dependence of the reaction rate in Arrhenius form $\boldsymbol{k\sim}\boldsymbol{e}^{\boldsymbol{-}\frac{\boldsymbol{E}}{\boldsymbol{RT}}}$, the condition for temperature-compensated oscillations (TCOs) is mathematically given by the following balance:

$\frac{1}{P}\frac{dP(T)}{dT}=\sum_{i} \frac{\partial lnP}{\partial{lnk}_{i}}\frac{dlnk_{i}}{dT}=\frac{1}{RT^{2}}\sum_{i} C_{i}E_{i}=0,$ (S1)

or simply,

$\sum_{i} C_{i}E_{i}=0$, (S2)

where *P* and *T* are the oscillation period and temperature, respectively, and *k_i_* is the reaction rate of the *i-*th reaction step with activation energy *E_i_*. $C_{i}\equiv\frac{\partial lnP}{\partial{lnk}_{i}}$ is the control coefficient, which is the sensitivity of the oscillation period to changes in rate $k_{i}$. The balance principle requires that activation energies $E_{i}$ and sensitivities $C_{i}$ satisfy Eq. (1) over the physiological temperature range. Generally, the control coefficient $C_{i}$ can depend on temperature, whereas the activation energy $E_{i}$ does not change. These feature complicate the analysis of temperature compensation.

**The TCO mechanism for motif C.** The dynamical model for motif C is given by the following equations:

$\frac{dx_{1}}{dt}=\frac{v_{11}{(\frac{x_{1}}{K_{11}})}^{n}}{1+{(\frac{x_{1}}{K_{11}})}^{n}+{(\frac{x_{2}}{K_{12}})}^{n}}+\delta_{1}-r_{1}x_{1}$ (S4)

$\frac{dx_{2}}{dt}=\frac{v_{21}{(\frac{x_{1}}{K_{21}})}^{n}}{1+{(\frac{x_{1}}{K_{21}})}^{n}}+\delta_{2}-r_{2}x_{2}$ (S5)

For spontaneous oscillations as demonstrated in S6 Fig, a complete cycle can be roughly divided into four stages according to the dynamical concentration of *x*_2_: the ascent phase $\tau_{1}$, during which *x*_2_ increases from 10% to 90% of its peak concentration (equivalent to approximately $\frac{v_{21}}{r_{2}}$); a short rising-to-falling transition phase $\tau_{2}$, where *x*_2_ turns from growth to decay; a descent phase $\tau_{3}$, during which the concentration *x*_2_ falls from 90% to 10% of the peak value; and the final transient phase $\tau_{4}$, in which the concentration trend reverses from decay to growth. The trajectory dwells for a much longer time in the ascent and descent phases than in the two transient phases; therefore $\tau_{1}$+$\tau_{3}$ contribute the most and determine the oscillation period. The ascent and descent periods $\tau_{1}$ and $\tau_{3}$ can be estimated quantitatively as shown in the following.

The basal rates $\delta_{1}, \delta_{2}$ are small and therefore can be neglected for approximation. Consider the situation in which $K_{21}$ is not large (this is the case in our simulations). As $x_{1}$ in the ascending phase takes generally larger values, the condition ${(\frac{x_{1}}{K_{21}})}^{n}\gg1$is approximately satisfied. In the ascending phase, Eq. (S5) can be simplified to the following form:

$\frac{dx_{2}}{dt}\approx v_{21}-r_{2}x_{2}$ (S6)

Let *a* and *b* denote 10% and 90% of the peak value of $x_{2}$, respectively. The time span of the ascending phase can be estimated as:

$\tau_{1}=\int_{a}^{b} \frac{dx_{2}}{v_{21}-r_{2}x_{2}}=\frac{1}{r_{2}}ln\left| \frac{1-\frac{r_{2}}{v_{21}}a}{1-\frac{r_{2}}{v_{21}}b} \right|$ (S7)

As $\frac{v_{21}}{r_{2}}$ is the peak value of $x_{2}$, $\tau_{1}\sim\frac{1}{r_{2}}ln9$ indicates that the ascending phase is primarily determined by the decay rate $r_{2}$. In the descending phase $x_{1}$ takes very small values, and therefore${(\frac{x_{1}}{K_{21}})}^{n}\ll$1 is satisfied. Equation (S5) could thus be reduced simply to the following:

$\frac{dx_{2}}{dt}\approx-r_{2}x_{2}$ (S8)

Once again, the time period of the descending phase is also $\tau_{3}\sim\frac{1}{r_{2}}ln9$. The time period for the two transition phases is relatively rather small and could be considered constant. Rough estimates indicate that the oscillation period is dominated mainly by the decay rate of $x_{2}$. Good TCOs in Motif C can be achieved by an insensitive dependence of $r_{2}$ on temperature. This is equivalent to a low activation energy for rate constant $r_{2}$, which depends on temperature in Arrhenius form. Therefore, the key for motif C to achieve TCOs is that the oscillation period is determined by those rates that are robust against temperature changes and is insensitive to other rates that might vary significantly with temperature. This mechanism for TCOs is abundant in more complex topologies, as demonstrated in 5S Fig.

**The TCO mechanism for motif B.** The ODEs describing the dynamical behaviors of motif B take the following form:

$\left\{ \begin{matrix} \frac{dx_{1}}{dt}=\frac{v_{1}}{1+{(\frac{x_{2}}{K_{12}})}^{n}}+\delta_{1}-r_{1}x_{1} \\ \frac{dx_{2}}{dt}=\frac{v_{2}}{1+{(\frac{x_{23}}{K_{23}})}^{n}}+\delta_{2}-r_{2}x_{2} \\ \frac{dx_{3}}{dt}=\frac{v_{3}}{1+{(\frac{x_{31}}{K_{31}})}^{n}}+\delta_{3}-r_{3}x_{3} \end{matrix} \right.$ (S9)

When the basal expression level and equilibrium constants are sufficiently small, that is, $\delta_{i}\approx0, and\left( \frac{x_{1}}{K_{21}} \right)^{n}\ll1, i=1, 2, 3$, the above equation can be simplified as follows:

$\left\{ \begin{matrix} \frac{dx_{1}}{dt}=v_{1}{(\frac{K_{12}}{x_{2}})}^{n}-r_{1}x_{1} \\ \frac{dx_{2}}{dt}=v_{2}{(\frac{K_{23}}{x_{3}})}^{n}-r_{2}x_{2} \\ \frac{dx_{3}}{dt}=v_{3}{(\frac{K_{31}}{x_{1}})}^{n}-r_{3}x_{3} \end{matrix} \right.$ (S10)

The equations can be reduced and rewritten with rescaled variables, $u_{1}=\alpha x_{1}, u_{2}=\beta x_{2}, u_{3}=\gamma x_{3}$, as in the following form:

$\left\{ \begin{matrix} \frac{du_{1}}{dt}={u_{2}}^{-n}-{r_{1}u}_{1} \\ \frac{du_{2}}{dt}={u_{3}}^{-n}-r_{2}u_{2} \\ \frac{du_{3}}{dt}={u_{3}}^{-n}-{r_{3}u}_{3} \end{matrix} \right.$ (S11)

Where $\alpha$, $\beta$ and $\gamma$ are defined as:

$\alpha={v_{1}}^{-\frac{1}{28}}{v_{2}}^{\frac{3}{28}}{v_{3}}^{-\frac{9}{28}}{K_{1}}^{-\frac{27}{28}}{K_{2}}^{-\frac{3}{28}}{K_{3}}^{\frac{9}{28}}$ (S12)

$\beta={v_{1}}^{-\frac{9}{28}}{v_{2}}^{-\frac{1}{28}}{v_{3}}^{\frac{3}{28}}{K_{1}}^{\frac{9}{28}}{K_{2}}^{-\frac{27}{28}}{K_{3}}^{-\frac{3}{28}}$ (S13)

$\gamma={v_{1}}^{\frac{3}{28}}{v_{2}}^{-\frac{9}{28}}{v_{3}}^{-\frac{1}{28}}{K_{1}}^{-\frac{3}{28}}{K_{2}}^{\frac{9}{28}}{K_{3}}^{-\frac{27}{28}}$ (S14)

($n=3$)

For oscillations described by Eqs. S11, the oscillation period depends obviously on only three constants: $r_{1}$, $r_{2}$ and $r_{3}.$ When the temperature is varied, the other constants are still affected, but they change only the amplitude of the oscillation because they have been absorbed into the rescaled variables. If the degradation rates $r_{1}$, $r_{2}$ and $r_{3}$ change rarely with very small activation energies, the oscillation period will be independent of changes in temperature. Based on this mechanism, motif B has more chances than motifs A, C, and D to find appropriate parameter combinations in the random sampling to achieve a TCO. This can be seen in Fig 2a, where the q-value for motif B is the highest among all simplest motifs. However, the feature of rescaled variables is lost when motif B is appended with additional regulations. Consequently, topologies based on motif B, such as motif combinations of AB in Fig 2b, are rare in robust TCO networks. Combinations of BD, BCD, ABD, and ABCD do not occur because the components B and D conflict with each other.

**Parameters for motifs A, B, C and D depicted in Fig 4**. The data are obtained by sampling in our calculations to achieve temperature-compensated oscillation (the unit of activation energy is ${KJ}/{mol}$):

**Motif A:**

Panel a_1_ in Fig 4:

| $v_{12} (a.u.)$ | 75.1373 | $E_{v12} (KJ/mol)$ | 91.3175 |
| --- | --- | --- | --- |
| $v_{2} (a.u.)$ | 4.4017 | $E_{v2} (KJ/mol)$ | 21.5105 |
| $v_{31} (a.u.)$ | 32.3268 | $E_{v31}(KJ/mol)$ | 70.2810 |
| $r_{1} (a.u.)$ | 2.3373 | $E_{r1}(KJ/mol)$ | 27.2672 |
| $r_{2} (a.u.)$ | 3.4795 | $E_{r2}(KJ/mol)$ | 4.5133 |
| $r_{3} (a.u.)$ | 2.5372 | $E_{r3}(KJ/mol)$ | 5.9500 |
| $K_{12} (a.u.)$ | 1.1751 | $K_{23} (a.u.)$ | 0.4789 |
| $K_{31} (a.u.)$ | 0.5592 |  |  |

Panel a_2_ in Fig 4:

| $v_{12} (a.u.)$ | 20.2075 | $E_{v12} (KJ/mol)$ | 22.0466 |
| --- | --- | --- | --- |
| $v_{2} (a.u.)$ | 32.2602 | $E_{v2} (KJ/mol)$ | 6.4342 |
| $v_{31} (a.u.)$ | 4.3018 | $E_{v31}(KJ/mol)$ | 54.0131 |
| $r_{1} (a.u.)$ | 0.7295 | $E_{r1}(KJ/mol)$ | 44.9472 |
| $r_{2} (a.u.)$ | 2.1761 | $E_{r2}(KJ/mol)$ | 6.1090 |
| $r_{3} (a.u.)$ | 4.6461 | $E_{r3}(KJ/mol)$ | 7.5286 |
| $K_{12} (a.u.)$ | 1.2560 | $K_{23} (a.u.)$ | 0.0474 |
| $K_{31} (a.u.)$ | 0.4205 |  |  |

**Motif B:**

Panel b_1_ in Fig 4:

| $v_{1} (a.u.)$ | 72.0327 | $E_{v1} (KJ/mol)$ | 53.2876 |
| --- | --- | --- | --- |
| $v_{2} (a.u.)$ | 54.4270 | $E_{v2} (KJ/mol)$ | 40.7005 |
| $v_{3} (a.u.)$ | 7.7616 | $E_{v3}(KJ/mol)$ | 58.5484 |
| $r_{1} (a.u.)$ | 3.2657 | $E_{r1}(KJ/mol)$ | 13.9308 |
| $r_{2} (a.u.)$ | 0.7077 | $E_{r2}(KJ/mol)$ | 40.1097 |
| $r_{3} (a.u.)$ | 0.5895 | $E_{r3}(KJ/mol)$ | 20.7207 |
| $K_{12} (a.u.)$ | 1.2442 | $K_{23} (a.u.)$ | 0.8975 |
| $K_{31} (a.u.)$ | 0.1225 |  |  |

Panel b_2_ in Fig 4:

| $v_{1} (a.u.)$ | 76.3547 | $E_{v1} (KJ/mol)$ | 97.3001 |
| --- | --- | --- | --- |
| $v_{2} (a.u.)$ | 2.6878 | $E_{v2} (KJ/mol)$ | 93.8843 |
| $v_{3} (a.u.)$ | 35.6883 | $E_{v3}(KJ/mol)$ | 41.8608 |
| $r_{1} (a.u.)$ | 1.0140 | $E_{r1}(KJ/mol)$ | 5.6923 |
| $r_{2} (a.u.)$ | 0.3062 | $E_{r2}(KJ/mol)$ | 23.7395 |
| $r_{3} (a.u.)$ | 0.7384 | $E_{r3}(KJ/mol)$ | 29.8998 |
| $K_{12} (a.u.)$ | 1.0288 | $K_{23} (a.u.)$ | 3.3588 |
| $K_{31} (a.u.)$ | 3.5744 |  |  |

**Motif C:**

Panel c_1_ in Fig 4:

| $v_{11} (a.u.)$ | 58.3185 | $E_{v11} (KJ/mol)$ | 95.7044 |
| --- | --- | --- | --- |
| $v_{21} (a.u.)$ | 22.9047 | $E_{v21} (KJ/mol)$ | 61.9621 |
| $r_{1} (a.u.)$ | 1.3313 | $E_{r1}(KJ/mol)$ | 23.0470 |
| $r_{2} (a.u.)$ | 0.6190 | $E_{r2}(KJ/mol)$ | 31.4024 |
| $K_{11} (a.u.)$ | 0.0773 | $K_{12} (a.u.)$ | 0.0293 |
| $K_{21} (a.u.)$ | 0.9872 |  |  |

Panel c_2_ in Fig 4:

| $v_{11} (a.u.)$ | 15.5317 | $E_{v11} (KJ/mol)$ | 49.7462 |
| --- | --- | --- | --- |
| $v_{21} (a.u.)$ | 93.3070 | $E_{v21} (KJ/mol)$ | 8.2498 |
| $r_{1} (a.u.)$ | 1.6079 | $E_{r1}(KJ/mol)$ | 11.7129 |
| $r_{2} (a.u.)$ | 0.1387 | $E_{r2}(KJ/mol)$ | 10.7176 |
| $K_{11} (a.u.)$ | 0.0100 | $K_{12} (a.u.)$ | 0.0583 |
| $K_{21} (a.u.)$ | 0.9734 |  |  |

**Motif D:**

Panel d_1_ in Fig 4:

| $v_{11} (a.u.)$ | 59.8371 | $E_{v11} (KJ/mol)$ | 91.0139 |
| --- | --- | --- | --- |
| $v_{12} (a.u.)$ | 2.7900 | $E_{v12} (KJ/mol)$ | 4.9611 |
| $v_{21} (a.u.)$ | 2.2976 | $E_{v21} (KJ/mol)$ | 44.1693 |
| $r_{1} (a.u.)$ | 0.2757 | $E_{r1}(KJ/mol)$ | 42.7280 |
| $r_{2} (a.u.)$ | 0.1234 | $E_{r2}(KJ/mol)$ | 31.8613 |
| $K_{11} (a.u.)$ | 0.3839 | $K_{12} (a.u.)$ | 0.0130 |
| $K_{21} (a.u.)$ | 56.3680 |  |  |

Panel d_2_ in Fig 4:

| $v_{11} (a.u.)$ | 88.2806 | $E_{v11} (KJ/mol)$ | 80.3749 |
| --- | --- | --- | --- |
| $v_{12} (a.u.)$ | 2.4901 | $E_{v12} (KJ/mol)$ | 54.2136 |
| $v_{21} (a.u.)$ | 5.6915 | $E_{v21} (KJ/mol)$ | 35.7226 |
| $r_{1} (a.u.)$ | 8.4083 | $E_{r1}(KJ/mol)$ | 43.1770 |
| $r_{2} (a.u.)$ | 0.2053 | $E_{r2}(KJ/mol)$ | 13.3548 |
| $K_{11} (a.u.)$ | 0.0206 | $K_{12} (a.u.)$ | 0.0113 |
| $K_{21} (a.u.)$ | 9.4021 |  |  |

**Parameters for circuits depicted in Fig 6b**.

| $v_{11} (a.u.)$ | 58.3185 | $E_{v11} (KJ/mol)$ | 95.7044 |
| --- | --- | --- | --- |
| $v_{21} (a.u.)$ | 22.9047 | $E_{v21} (KJ/mol)$ | 61.9621 |
| $r_{1} (a.u.)$ | 1.3313 | $E_{r1}(KJ/mol)$ | 23.0470 |
| $r_{2} (a.u.)$ | 0.6190 | $E_{r2}(KJ/mol)$ | 31.4024 |
| $K_{11} (a.u.)$ | 0.0773 | $K_{12} (a.u.)$ | 0.0293 |
| $K_{21} (a.u.)$ | 0.9872 |  |  |

**Parameters for circuits depicted in Fig 6c**.

| $v_{11} (a.u.)$ | 15.5317 | $E_{v11} (KJ/mol)$ | 49.7462 |
| --- | --- | --- | --- |
| $v_{21} (a.u.)$ | 93.3070 | $E_{v21} (KJ/mol)$ | 8.2498 |
| $r_{1} (a.u.)$ | 1.6079 | $E_{r1}(KJ/mol)$ | 11.7129 |
| $r_{2} (a.u.)$ | 0.1387 | $E_{r2}(KJ/mol)$ | 10.7176 |
| $K_{11} (a.u.)$ | 0.0100 | $K_{12} (a.u.)$ | 0.0583 |
| $K_{21} (a.u.)$ | 0.9734 |  |  |

**Dynamical ODEs for motif A**:

$$\left\{ \begin{matrix} \frac{dx_{1}}{dt}=\frac{v_{12}{(\frac{x_{2}}{K_{12}})}^{n}}{1+{(\frac{x_{2}}{K_{12}})}^{n}}+\delta_{1}-r_{1}x_{1} \\ \frac{dx_{2}}{dt}=\frac{v_{2}}{1+{(\frac{x_{3}}{K_{23}})}^{n}}+\delta_{2}-r_{2}x_{2} \\ \frac{dx_{3}}{dt}=\frac{v_{31}{(\frac{x_{1}}{K_{31}})}^{n}}{1+{(\frac{x_{1}}{K_{31}})}^{n}}+\delta_{3}-r_{3}x_{3} \end{matrix} \right.$$

**Dynamical ODEs for motif D**:

$$\left\{ \begin{aligned} \frac{dx_{1}}{dt}=\frac{v_{11}\left( \frac{x_{1}}{K_{11}} \right)^{n}+v_{12}\left( \frac{x_{2}}{K_{12}} \right)^{n}}{1+\left( \frac{x_{1}}{K_{11}} \right)^{n}+\left( \frac{x_{2}}{K_{12}} \right)^{n}v_{12}\left( \frac{x_{2}}{K_{12}} \right)^{n}}+\delta_{1}-r_{1}x_{1} \\ \frac{dx_{2}}{dt}=\frac{v_{21}{(\frac{x_{1}}{K_{21}})}^{n}}{1+{(\frac{x_{1}}{K_{21}})}^{n}}+\delta_{2}-r_{2}x_{2} \end{aligned} \right.$$

**Dynamical ODES for the motif combination A+C in S3 Fig**:

$$\left\{ \begin{matrix} \frac{dx_{1}}{dt}=\frac{v_{11}{(\frac{x_{1}}{K_{11}})}^{n}}{1+{(\frac{x_{1}}{K_{11}})}^{n}+{(\frac{x_{2}}{K_{12}})}^{n}+{(\frac{x_{3}}{K_{13}})}^{n}}+\delta_{1}-r_{1}x_{1} \\ \frac{dx_{2}}{dt}=\frac{v_{21}{(\frac{x_{1}}{K_{21}})}^{n}}{1+{(\frac{x_{1}}{K_{21}})}^{n}}+\delta_{2}-r_{2}x_{2} \\ \frac{dx_{3}}{dt}=\frac{v_{32}{(\frac{x_{2}}{K_{32}})}^{n}}{1+{(\frac{x_{2}}{K_{32}})}^{n}}+\delta_{3}-r_{3}x_{3} \end{matrix} \right.$$

**Dynamical ODES for the motif combination B+C in S3 Fig**:

$$\left\{ \begin{matrix} \frac{dx_{1}}{dt}=\frac{v_{11}{(\frac{x_{1}}{K_{11}})}^{n}}{1+{(\frac{x_{1}}{K_{11}})}^{n}+{(\frac{x_{2}}{K_{12}})}^{n}+{(\frac{x_{3}}{K_{13}})}^{n}}+\delta_{1}-r_{1}x_{1} \\ \frac{dx_{2}}{dt}=\frac{v_{21}{(\frac{x_{1}}{K_{21}})}^{n}}{1+{(\frac{x_{1}}{K_{21}})}^{n}+{(\frac{x_{3}}{K_{23}})}^{n}}+\delta_{2}-r_{2}x_{2} \\ \frac{dx_{3}}{dt}=\frac{v_{3}}{1+{(\frac{x_{1}}{K_{31}})}^{n}}+\delta_{3}-r_{3}x_{3} \end{matrix} \right.$$

**Parameters for Motif C depicted in S6 Fig.**

| $v_{11} (a.u.)$ | 57.4523 | $E_{v11} (KJ/mol)$ | 99.6706 |
| --- | --- | --- | --- |
| $v_{21} (a.u.)$ | 38.1739 | $E_{v21} (KJ/mol)$ | 96.8089 |
| $r_{1} (a.u.)$ | 0.8537 | $E_{r1}(KJ/mol)$ | 12.5766 |
| $r_{2} (a.u.)$ | 0.2685 | $E_{r2}(KJ/mol)$ | 11.7727 |
| $K_{11} (a.u.)$ | 0.0264 | $K_{12} (a.u.)$ | 0.0506 |
| $K_{21} (a.u.)$ | 1.8065 |  |  |
